# Improving the estimation accuracy of rapeseed leaf photosynthetic characteristics under salinity stress using continuous wavelet transform and successive projections algorithm

**DOI:** 10.3389/fpls.2023.1284172

**Published:** 2023-11-14

**Authors:** Jingang Wang, Tian Tian, Haijiang Wang, Jing Cui, Xiaoyan Shi, Jianghui Song, Tiansheng Li, Weidi Li, Mingtao Zhong, Wenxu Zhang

**Affiliations:** ^1^ College of Agriculture, Shihezi University, Shihezi, China; ^2^ Key Laboratory of Oasis Ecological Agriculture of Xinjiang Production and Construction Corps, Shihezi University, Shihezi, Xinjiang, China

**Keywords:** photosynthesis, continuous wavelet transformation, partial least squares regression, support vector machines, brassic anapus

## Abstract

Soil salinization greatly restricts crop production in arid areas for salinity stress can inhibit crop photosynthesis and growth. Chlorophyll fluorescence and photosynthetic gas exchange (CFPGE) parameters are important indicators of crop photosynthesis and have been widely used to evaluate the impacts of salinity stress on crop photosynthesis and growth. Remote sensing technology can quickly and non-destructively obtain crop information under salinity stress, however, at present, the distribution of spectral features of CFPGE parameters in different regions is still unclear. In this study (2019-2020), under salinity stress conditions, the spectral data of rapeseed leaves were acquired and the CFPGE parameters were simultaneously determined. Then, continuous wavelet transformation (CWT) and standard normal variate (SNV) transformation were utilized to preprocess the raw spectral data. After that, a CFPGE parameter estimation model was constructed by using the partial least squares regression (PLSR) algorithm and the support vector machines (SVM) algorithm based on the spectral features in the red region (600-800 nm) and those in the red, blue-green (350-600 nm), and near-infrared (800-2500 nm) regions. The results showed that the spectral features of CFPGE parameters could be extracted by successive projections algorithm (SPA) based on the CWT preprocessing. The CFPGE parameter estimation model constructed based on the spectral features in the red region (675 nm, 680 nm, 688 nm, 749 nm, and 782 nm) had the highest Fv/Fm estimation accuracy on day 30, with R^2^c, R^2^p, and RPD of 0.723, 0.585, and 1.68, respectively. Based on this, the spectral features (578 nm, 976 nm, 1088 nm, 1476 nm, and 2250 nm) in the blue-green and near-infrared regions were added in the variables for modeling, which significantly improved the accuracy and stability of the model, with R^2^c, R^2^p, and RPD of 0.886, 0.815, and 2.58, respectively. Therefore, the fusion of the spectral features in the red, blue-green, and near-infrared regions could improve the estimation accuracy of rapeseed leaf CFPGE parameters. This study will provide technical reference for rapid estimation of photosynthetic performance of crops under salinity stress in arid and semi-arid areas.

## 1 Introduction

Soil salinization is a global environmental problem (Zhang et al., 2014; Wang et al., 2020; Zhu et al., 2023)). Currently, about 25% of the world’s arable land is affected by soil salinization, causing salinity stress to crops (Qadir et al., 2014). Salinity stress exerts multifaceted effects on crop growth, especially photosynthesis (Ben-asher et al., 2006). Under salinity stress, the chloroplast ultrastructure is destructed, and the photochemical efficiency of photosystem II (PSII) is reduced, leading to decreased photosynthetic rate of crops. This eventually suppresses crop growth (Fariduddin, 2013; Banakar et al., 2022).

Chlorophyll fluorescence and photosynthetic gas exchange (CFPGE) parameters reflect crop photosynthetic performance, and are key indicators to evaluate the response of crop photosynthesis and physiological and biochemical activities to environmental stress. Under salinity stress, the CFPGE parameters of crops change prior to chlorophyll content and salt ions, that is, CFPGE parameters are sensitive to salinity stress (Zarco-Tejada et al., 2003; Liu et al., 2013; Hniličková et al., 2017). Therefore, CFPGE parameters can be used to monitor whether the photosynthetic system of crops is damaged by salinity stress. These parameters are of great importance for analyzing the response mechanism of crops to salinity stress (Baker and Rosenqvist, 2004). Traditionally, the CFPGE parameters of crops was mostly non-destructively monitored using portable devices. However, the device operation is complicated and time-consuming. Especially, it requires shading treatment (dark adaptation) and other treatments before measurement. All these factors limit its large scale applications (Li, 2021).

Hyperspectral imaging technique, a new remote sensing technique, can quickly, accurately, and non-destructively monitor the photosynthetic and chlorophyll fluorescence signal changes and the photosynthetic performance of crops over a large area (Hamzeh et al., 2013; Tirado et al., 2020). Under external stresses such as salinity, drought, pests and diseases, etc., crop leaf spectral reflectance changes, reflecting the changes of physical and biochemical components within crops. This is the direct basis for spectral detection of the effects of environmental stresses on crops (Ashourloo et al., 2014; Huang et al., 2019). Previous studies have found that chlorophyll fluorescence parameters have two peaks (690 nm and 740 nm) in 600-800 nm, which can be used for the rapid spectral detection of chlorophyll fluorescence (Buschmann et al., 2000; Zhang et al., 2012). For example, Zarco-Tejada et al. (2000) constructed a hyperspectral vegetation index based on the 600-800 nm region, and found that the vegetation index based on R680/R630 accurately estimated the CFPGE parameters. Some studies have also achieved accurate estimation of CFPGE parameters in Phyllostachysacuta (Wu et al., 2014), wheat (Zhu et al., 2007), corn (Tan et al., 2012), and cotton (Xue et al., 2013) based on the red region. Notably, most of the above studies used wavelengths in the red region (600-800 nm) to monitor crop leaf CFPGE parameters. However, the blue-green (350-600 nm) and near-infrared (800-2500 nm) regions may also contain spectral information closely related to crop CFPGE parameters, which deserves further exploration (Magney et al., 2014; Porcar-Castell et al., 2014).

The chlorophyll fluorescence signal peaks are weak in the blue-green and near-infrared regions, making it difficult to extract spectral features (Mohammed et al., 2019). Currently, many studies eliminate background interference and improve the correlations between crop physiological components and spectra by spectral transformation (Zheng et al., 2021). Continuous wavelet transformation (CWT) can capture subtle changes in reflectance and highlight the weak spectral features of crops (Liu et al., 2020; Zhang et al., 2020). Analysis based on full spectrum may cause some redundancies and collinearity (Liu et al., 2020). Variable optimization by feature extraction algorithms such as Monte Carlo uninformative variables elimination (MC-UVE) (Li et al., 2017) and successive projections algorithm (SPA) can greatly reduce redundant information and minimize collinear variables (Galvo et al., 2008; Jia et al., 2019).

In summary, soil salinization affects crop photosynthesis, and CFPGE parameters can accurately characterize crop photosynthetic capacity and PSII damage under salinity stress. At present, the response mechanism of crop CFPGE parameters to salinity stress is not clear, and whether the spectral features in the blue-green and near-infrared regions can improve the accuracy of spectral estimation of crop leaf CFPGE parameters needs to be further explored. Therefore, in this study, the effects of salinity stress on rapeseed leaf photosynthesis were explored, and the spectral data of rapeseed leaves were acquired. After preprocessing the spectral data using the CWT, the spectral features of rapeseed leaf CFPGE parameters in the blue-green, red, and near-infrared regions under salinity stress were selected by SPA. Finally, CFPGE parameter estimation models based on PLSR and SVM were constructed. The objectives of this study were to explore: (1) the response mechanism of rapeseed CFPGE parameters to salinity stress; (2) the distribution of the spectral features of CFPGE parameters in the blue-green, red, and near-infrared regions; and (3) the effects of feature selection and different modeling strategies on the accuracy of the CFPGE parameter estimation model. This study will provide technical reference for accurate, rapid, and non-destructive monitoring of photosynthetic performance of crops under salinity stress in arid and semi-arid areas.

## 2 Materials and methods

### 2.1 Experimental site

The experiment was conducted from October to December 2019 and from March to May 2020 at the Experimental Station of Shihezi University (86°3′ N, 44°18′ E, a.s.l. 428 m) in Xinjiang Uygur Autonomous Region, China. The area has a temperate continental climate, with an annual average sunshine duration of 2725-2820 h, an annual average accumulated temperature (≥ 10 °C) of 3595 - 3729 °C, an annual average precipitation of 125.0-207.7 mm, and a frost-free period of 168-171 days. The soil was taken from an arable land (0-20 cm soil layer) in Yuephu County, Kashgar, Xinjiang, China (39°02′ N, 77°24′ E), and the soil type was gray desert soil. The soil pH measured with a PHS-P acidity meter (Leici, Shanghai, China) was 7.64. The soil organic matter content was 12.05 mg·kg^-1^. The soil total nitrogen content measured by a K9840 Kjeldahl analyzer (Qianjun, Shanghai, China) was 0.89 mg·kg^-1^. The soil available nitrogen content was 93.6 mg·kg^-1^. The soil available phosphorus content was 18.7 mg·kg^-1^. The soil available potassium content measured by the flame photometry (FP-6410, Xinyi Instruments., Shanghai, China) was 242 mg·kg^-1^. The soil salts were mainly chlorides, and the soil conductivity measured by a BPH-6600 conductivity meter (Bell, Dalian, China) was 1.15 g·kg^-1^. All above soil properties were measured according to the methods of Bao (2000).

### 2.2 Experimental design

Plump rapeseed seeds (variety Huayouza 62, a double-low rapeseed variety suitable for growing in northern China; provided by Huazhong Agricultural University) with consistent size were soaked in 70% alcohol for 30 s, disinfected with sodium hypochlorite for 10 min, and rinsed with distilled water 5 times. Then, the water on seed surface were absorbed using absorbent paper. After that, the seeds were sown in a dish containing humus and vermiculite (1: 1) (1 seed per hole, 3-5 cm in depth), and cultivated in an incubator (Ningbo Southeast Instrument Co., Ltd., China) (light intensity: 15000 lx; light/dark cycle: 14/10 hours; temperature: 23 ± 2°C under light and 18 ± 2°C in dark). When the seedlings had two leaves, seedlings of similar size and good growth status were transplanted to outdoor pots after removing the root zone soil. Three plants were planted in each pot (upper diameter: 25cm, bottom diameter: 20 cm, height: 30 cm).

According to the classification standard of salinized soil in Xinjiang, China (non-saline soils (0-3 g·kg^-1^), mildly saline soils (3-5 g·kg^-1^), moderately saline soils (5-10 g·kg^-1^), highly saline soils (10-20 g·kg^-1^) (Luo, 1985), soils were separately mixed with 0 (S0), 3.5 (S1), 5.5 (S2), and 7.5 (S3) g·kg^-1^ of NaCl, and filled in pots (5 kg soil per pot). Each group had 40 pots. Besides, 200 kg·ha^-1^ of urea (N, 46%), 90 kg·ha^-1^ of heavy superphosphate (P_2_O_5_, 46-54%), and 75 kg·ha^-1^ of potassium sulfate (K_2_O, 50%) were basally applied.

### 2.3 Data collection and indoor determination

#### 2.3.1 Data collection

The portable PSR-3500 visible-NIR spectrometer (Spectral Evolution Inc., Lawrence, MA, USA) with a wavelength range of 350~2500 nm was used to collect the rapeseed leaf spectra 10, 20, 30, and 40 days after transplanting. The spectrometer has three detectors: (a) A 512-element silicon photodiode array (spectral range: 350-1000 nm; resolution: 3.5 nm; interval: 1.5); (b) A 256-element InGaAs array (spectral range: 970-1910 nm; resolution: 7 nm; interval: 3.8); and (c) A 256-element InGaAs array (spectral range: 1900-2500 nm; resolution: 10 nm: interval: 2.5). The reflectance was resampled to 1 nm, and 2151 bands were output from 350 ~ 2500 nm. Spectral data were acquired on cloudless and windless days (Tian et al., 2022). The spectrometer was calibrated with a white plate every 10 samples. During spectral acquisition, the leaf clip connected to the spectrometer was used to acquire the spectral reflectance of the left, middle, and right parts of the leaves, and the average value was calculated to obtain the spectral reflectance (Tian et al., 2022). Finally, two hundred and forty leaf spectral data were collected for each sampling.

### 2.3.2 Determination of chlorophyll fluorescence and photosynthetic gas exchange parameters

Fully expanded leaves were selected for the determination of photosynthetic gas exchange parameters and chlorophyll fluorescence parameters 10, 20, 30, and 40 days after transplanting. At 9:00-11:00, the LI-6400 portable photosynthesis system (LI-COR, Lincoln, NE, USA) was used to determine the net photosynthetic rate (Pn), intercellular carbon dioxide concentration (Ci), stomatal conductance (gs), and transpiration rate (Tr) of the top four leaves of each plant. The light intensity was set to 1000 µmol·m^-2^·s^-1^, the CO_2_ concentration was 400 µmol·mol^-1^, and the temperature was 25 °C.

The chlorophyll fluorescence parameters were measured by a PAM-2500 portable instrument equipped with a 2030-B leaf-clip holder (Walz, Germany). Firstly, the steady state fluorescence yield (Fs) of rapeseed leaves under photoreaction was determined, and then a strong light (1200 µmol·m^-2^·s^-1^, pulse time: 0.8 s) was given to determine the maximum fluorescence yield (Fm′) and the minimum fluorescence (F0′). After the light transmission hole was closed for 30 min, a strong light was given, to measure the maximum fluorescence yield in the dark-adapted state (Fm), initial fluorescence (F0), and photosynthetically active radiation (PAR). Finally, the photochemical quenching coefficient (qP), non-photochemical quenching coefficient (NPQ), actual photochemical efficiency of PSII (ΦPSII), maximum photochemical efficiency of PSII (Fv/Fm), potential activity of PSII (Fv/F0), and electron transport rate (ETR) were calculated according to the following formulas.


(1) Fv/Fm=(Fm−F0)/Fm

(2) Fv/F0=(Fm−F0)/F0

(3) ΦPS II=(Fm'−Fs)/Fm'

(4) qp=(Fm'−Fs)/(Fm'−F0')

(5) NPQ=(Fm−Fm')/Fm'

(6) ETR=PAR×ΦPSII×0.84×0.5

### 2.4 Descriptive statistical analysis of photosynthetic gas exchange and chlorophyll fluorescence parameters of rapeseed leaves

**Figure 1:**
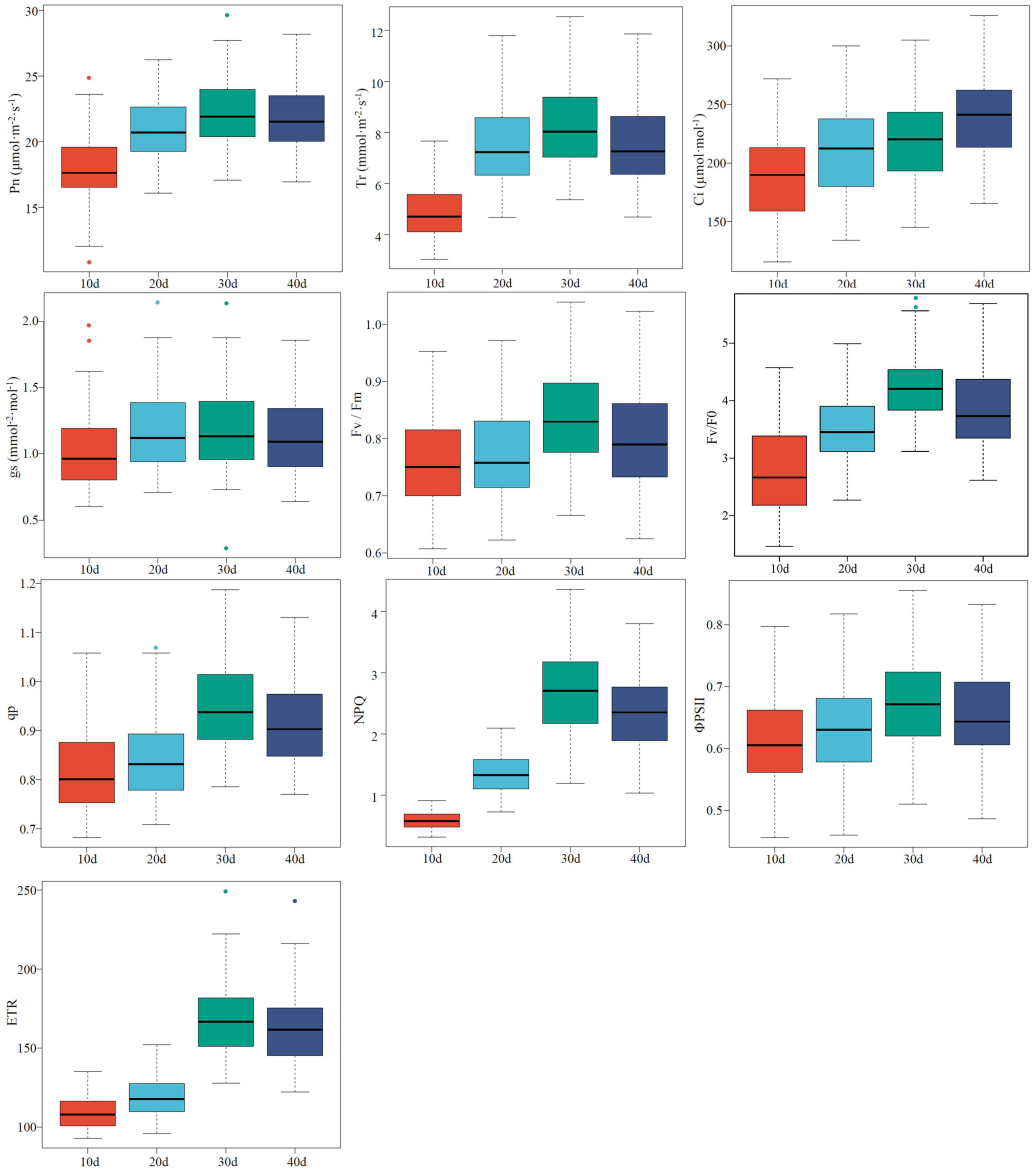
Descriptive statistical analysis of photosynthetic gas exchange and chlorophyll fluorescence parameters of rapeseed leaves. The line in the center of each box represents the median, the upper and lower boundaries of the extended line are the maximum and minimum values, respectively, and “●” represents an outlier.

The values of the CFPGE parameters were divided into three sub-classes from high to low, and the values with a large error were eliminated. Two-thirds of the samples were included into the modeling set and the left samples were included into the validation set (**Table S1**). The high degree of discreteness of each parameter indicates that the samples are sufficient and representative (**Figure 1**).

### 2.5 Spectral preprocessing

During spectral acquisition, the influence of environment and instrument is easy to cause a large amount of noise in the spectral data. To eliminate the noise and highlight the useful information in the spectral data, different preprocessings of the raw spectrum has been studied, such as SG smoothing, multivariate scattering correction (MSC), first derivative, etc. (Mahanti et al., 2020; Zhang et al., 2023). In this study, the raw spectra were preprocessed with continuous wavelet transform (CWT), which has great potential for extracting spectral information of CFPGE parameters. Besides, this study also compared CWT with the standard normal transform (SNV) with good performance in existing studies (Cheng et al., 2010). (**Figure S1**).

#### 2.5.1 Continuous wavelet transformation

Wavelet transform uses wavelet basis functions to decompose complex signals into wavelets of different scales (frequencies). It can extract weak information and highlight regional characteristics (Koger et al., 2003). The wavelet coefficients have two dimensions, namely the decomposition scale (i = 1, 2,…, m) and the band (j = 1, 2,…, n). That is, CWT converts one-dimensional hyperspectral reflectance into two-dimensional wavelet coefficients (Cheng et al., 2010).


(7) $$Wf(\text{a},b)=\int_{-\text{∞}}^{+\infty }f(\lambda ){\text{Ψ}}_{a,b}(\lambda )d\lambda$$

(8) $${\text{Ψ}}_{\left(a,b\right)}(\lambda )=\frac{1}{\sqrt{a}}\text{Ψ}(\frac{\lambda -b}{a})$$

where *Wf (a, b)* is wavelet coefficient, *f(λ)* is hyperspectral reflectance, *λ* is the spectral region of 350~2500 nm, and Ψ*_a,b_(λ)* is the wavelet basis function transformed by scale factor *a* and expansion factor *b*.

In this study, Gaus1 wavelet function was selected to carry out CWT and first derivative preprocessings. To reduce data redundancy, the decomposition scale of CWT was set as 2^1^ (CWT-1), 2^2^ (CWT-2),…, 2^10^ (CWT-10) (Yao et al., 2018).

#### 2.5.2 Standard normal variate (SNV) transformation

The SNV algorithm processes each spectrum based on the assumption that in each spectrum, the absorbance of the wavelengths are distributed in a certain rule (such as normal distribution) (Barnes et al., 1989). The essence is to normalize the raw spectral data, eliminate the constant offset by subtracting the average value of the whole spectrum, and then divide the standard deviation of the full spectra, to make the spectrum reach a similar proportion (Grisanti et al., 2018).


(9) $$x_{SNV}=\frac{x-\bar{x}}{\sqrt{\frac{\sum_{i=1}^{p}\left(x_{i}-\bar{x}\right)}{p-1}}}$$

where *x* is the raw spectrum of a sample, $\bar{x}$ is the average spectra of all wavelengths of the sample, i = 1, 2,…, p, and p is the number of wavelengths.

### 2.6 Successive projections algorithm

The SPA was used for feature extraction. The SPA compares the size of the vector by projecting the wavelength onto other wavelengths, takes the wavelength with the largest projection vector as the wavelength to be selected, and then selects the spectral feature based on the correction model. The SPA selects variables with with minimal redundancy and collinearity. The specific algorithm can be found in the reports of Galvo et al. (2008).

### 2.7 Modeling strategies

In this study, the CFPGE parameter estimation model was constructed based on the spectral features extracted from the red region and the spectral features extracted from the red, blue-green, and near-infrared regions, respectively. The modeling strategies employed were PLSR and SVM. PLSR processes data with high dimensional and multicollinearity by reducing collinear variables to non-correlated factors (latent variables, LV). Then, an estimation model with LVs as independent variables is established (Inoue et al., 2016).

SVM is superior to other methods in solving problems such as small sample size, nonlinearity, and multidimensionality. In this study, the Monte Carlo cross-validation was performed to optimize the penalty parameter c and reciprocal g of the radius of influence of the sample. The change ranges of the c and g were set to -1~1 (Hong et al., 2019). The optimal combination of c and g was selected based on the results of multiple cross-validations (Feng et al., 2018).

### 2.8 Model validation

The PLSR and SVM models were evaluated using coefficient of determination (R^2^), root mean squared error (RMSE), and residual prediction deviation (RPD). The larger the R^2^, the smaller the RMSE, the higher the prediction accuracy of the model. The smaller the RPD, the poorer robustness of the PLSR model. An increase of RPD value means the improvement of prediction accuracy (Chen et al., 2016).


(10) $$RMSE_{C}=\sqrt{\frac{1}{n_{c}}\sum_{i=1}^{n_{c}}(y_{ci}-{\hat{y}}_{ci}{)}^{2}}$$

(11) $$R_{c}^{2}=1-\frac{\sum_{i=1}^{n_{c}}{\left(y_{ci}-{\hat{y}}_{ci}\right)}^{2}}{\sum_{i=1}^{n_{c}}{\left(y_{ci}-{\bar{y}}_{c}\right)}^{2}}$$

(12) $$RMSE_{P}=\sqrt{\frac{1}{n_{p}}\sum_{i=1}^{n_{p}}{\left(y_{pi}-{\hat{y}}_{pi}\right)}^{2}}$$

(13) $$R_{p}^{2}=1-\frac{\sum_{i=1}^{n_{p}}{\left(y_{pi}-{\hat{y}}_{pi}\right)}^{2}}{\sum_{i=1}^{n_{p}}{\left(y_{pi}-{\bar{y}}_{p}\right)}^{2}}$$

(14) $$RPD=\frac{S_{D}}{RMSE_{CV}}$$

where ${\hat{y}}_{ci}$ is the predicted value of the i-th sample in the calibration set, *n_c_* is the number of samples in the calibration set, ${\bar{y}}_{c}$ is the average of the measured values for all samples in the calibration set, *y_pi_* is the measured value of the i-th sample in the validation set, ${\hat{y}}_{pi}$ is the predicted value of the i-th sample in the validation set, *n_p_* is the number of samples in the validation set, ${\bar{y}}_{p}$ is the average of the measured values for all samples in the validation set, *S_D_* is the standard deviation of the measured value for the sample in the validation set, and *RMSE_CV_* is the root mean square error of the cross-validation.

### 2.9 Data analysis

**Figure 2:**
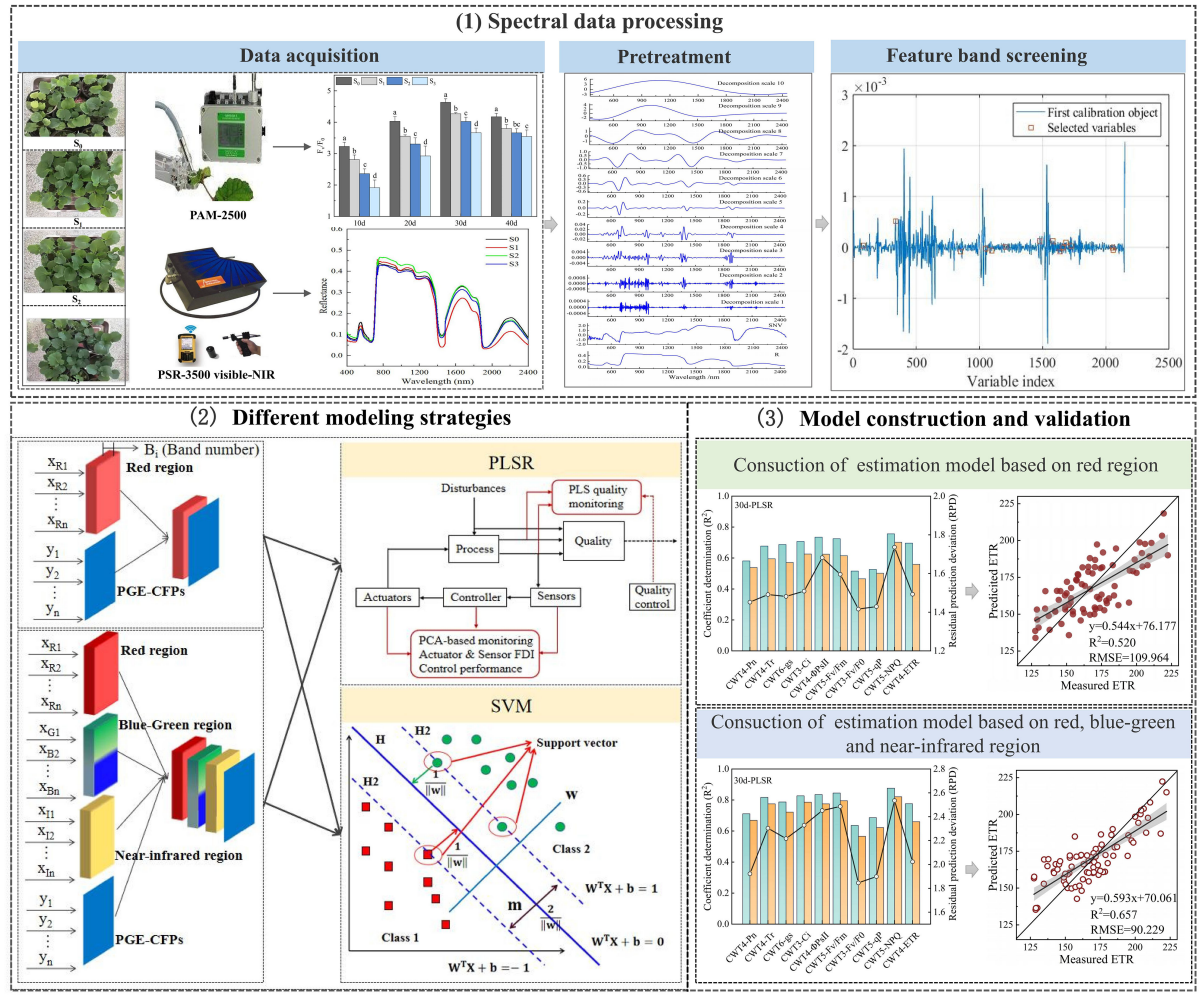
Construction of the estimation model of forage rape leaves CFPGE parameter under salinity stress.

Single factor analysis of variance (One-way ANOVA) was conducted using SPSS software version 21.0 (AMOS IBM, USA), and significance of the differences in means of the samples was analyzed using Duncan test at *p*< 0.05 (Clarke and Green, 1988). The CWT and SPA was performed using Matlab software version 2016a (MathWorks, Natick, MA, USA). The PLSR and SVM models were constructed using Unscramber X software version 10.1 (CAMO ASA, Trondheim, Norway). Graphics were drawn using Origin software version 2018 (Origin Lab, Massachusetts, USA). **Figure 2** shows the construction process of CFPGE parameter estimation model for rapeseed leaves under salinity.

## 3 Results

### 3.1 Effects of salinity stress on chlorophyll fluorescence and photosynthetic gas exchange parameter of rapeseed leaves

#### 3.1.1 Effects of salinity stress on photosynthetic gas exchange parameters in rapeseed leaves

**Figure 3:**
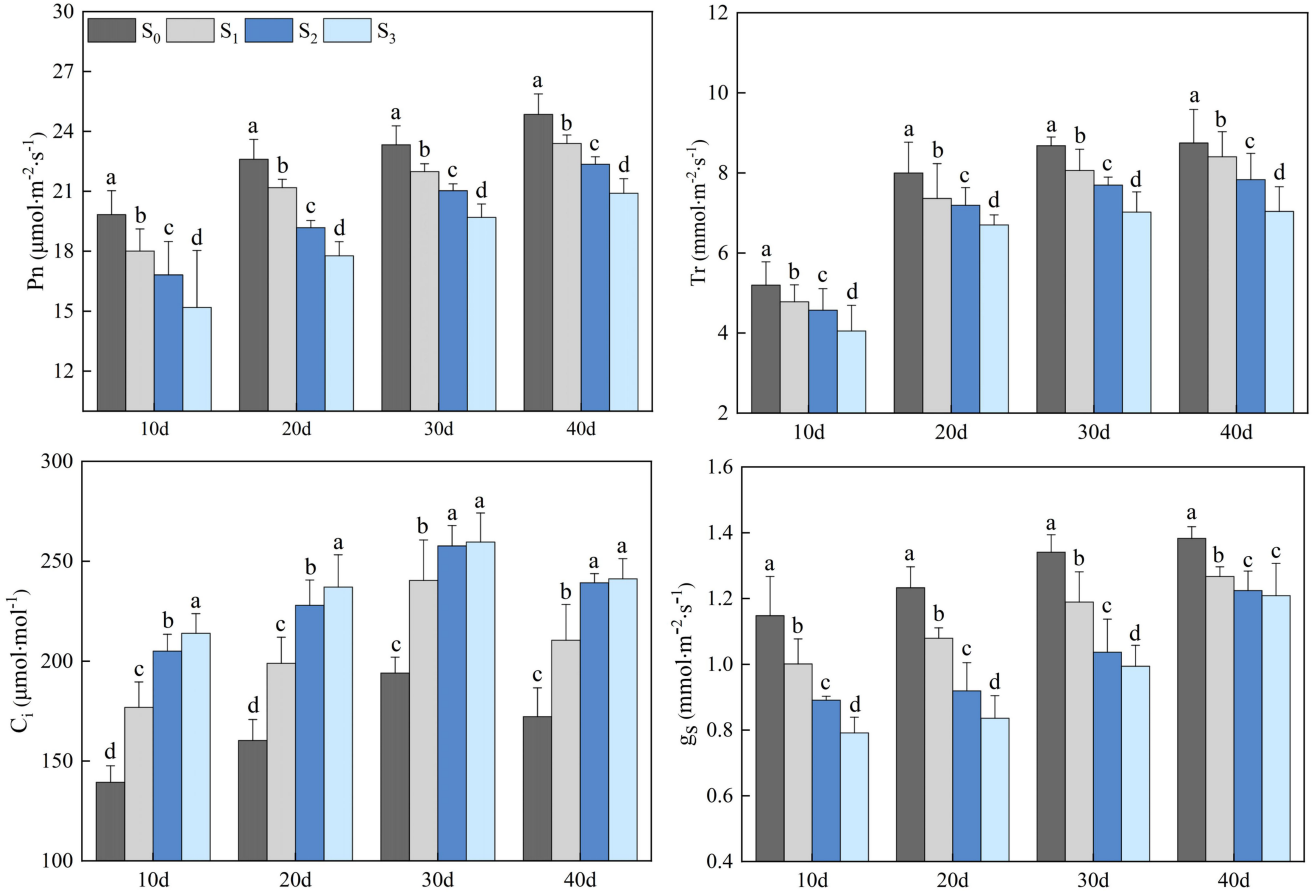
Changes in rapeseed leaf photosynthetic gas exchange parameters at different soil salinity levels. Different lowercase letters in the same column indicate significant difference between groups (*p*< 0.05).

Salinity treatments (S1, S2, and S3) significantly impacted the photosynthetic gas exchange parameters of rapeseed leaves (**Figure 3**). The Pn, Tr, and gs of rapeseed leaves in the S1, S2, and S3 groups gradually increased, and peaked on day 40. The Pn in the S1, S2, and S3 groups were 23.39, 22.36, and 20.91 µmol·m^-2^·s^-1^, respectively, which decreased by 5.87%, 11.18%, and 18.89%, compared with that in the S0 group (24.86 µmol·m^-2^·s^-1^) (*p*< 0.05). The Tr in the S1, S2, and S3 groups were 8.40, 7.83, and 7.04 mmol·m^-2^·s^-1^, respectively, which decreased by 4.17%, 11.75%, and 24.29% compared with that in the S0 group (8.75 mmol·m^-2^·s^-1^) (*p*< 0.05). The gs in the S1, S2, and S3 groups were 1.27, 1.22, and 1.21 mmol·m^-2^·s^-1^, respectively, which decreased by 8.66%, 13.11%, and 14.05% compared with that in the S0 group (1.38 mmol·m^-2^·s^-1^) (*p*< 0.05). The Ci in the S1, S2, and S3 groups increased first and then decreased, peaking on day 30. On day 30, the Ci in the S1, S2, and S3 groups were 240.39, 257.69, and 259.63 µmol·mol^-1^, respectively, which increased by 23.87%, 32.79%, and 33.81% compared with that in the S0 group (194.06 µmol·mol^-1^) (*p*< 0.05).

#### 3.1.2 Effects of salinity stress on chlorophyll fluorescence parameters of rapeseed leaves

**Figure 4:**
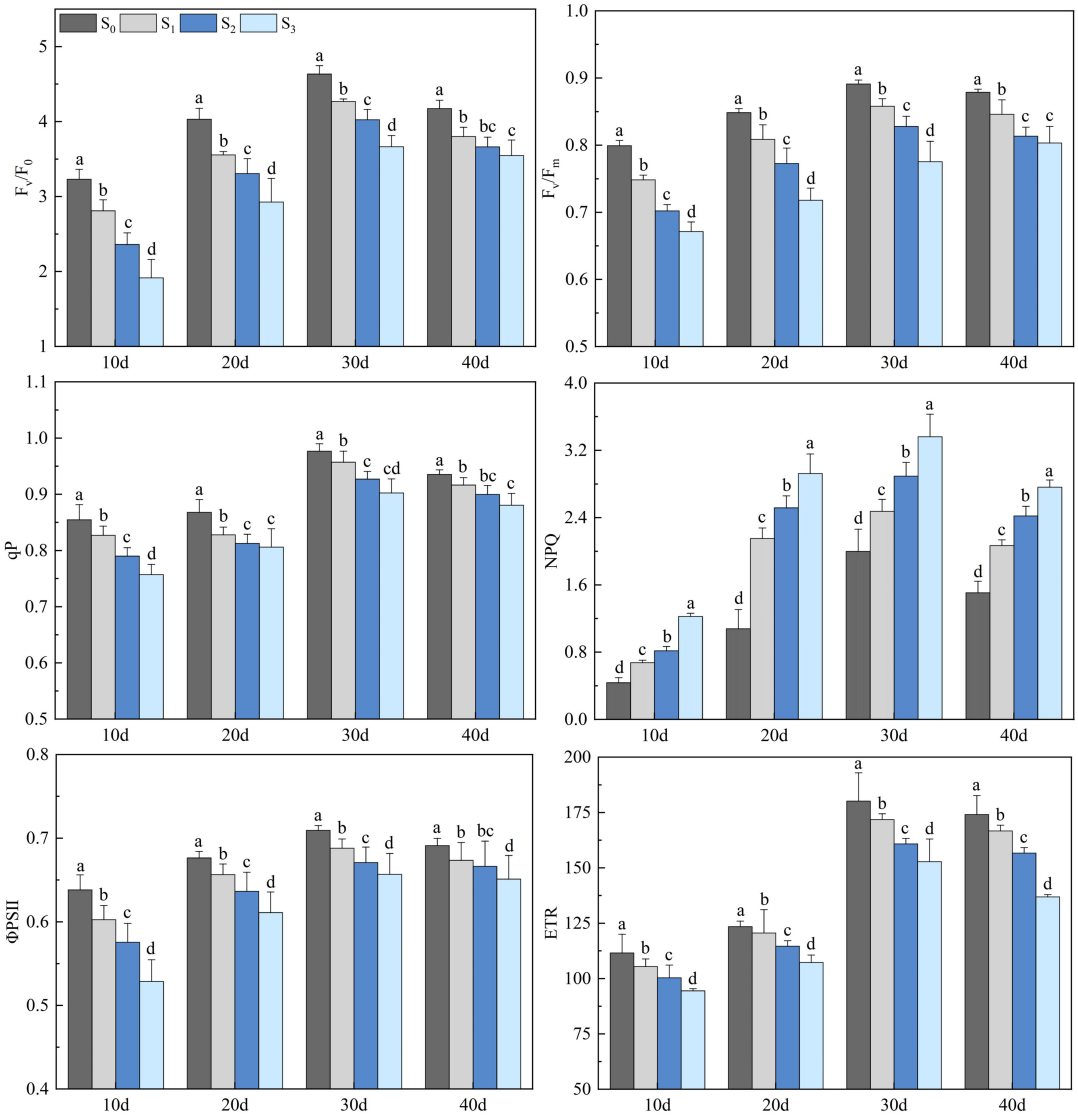
Changes in rapeseed leaf chlorophyll fluorescence parameters at different soil salinity levels. Different lowercase letters in the same column indicate significant difference between groups (*p*< 0.05).

Salinity treatments obviously impacted the chlorophyll fluorescence parameters of rapeseed leaves (**Figure 4**). The Fv/F0, Fv/Fm, qP, ΦPSII, and ETR of rapeseed leaves decreased with the increase of soil salinity (*p*< 0.05), while the NPQ showed an opposite trend (*p*< 0.05). The chlorophyll fluorescence parameters of rapeseed leaves in the S1, S2, and S3 groups increased first, peaked on day 30, and then decreased. On day 30, the Fv/F0 in the S1, S2, and S3 groups were 4.27, 4.03, and 3.66, respectively, which decreased by 9.74%, 13.93%, and 17.46% compared with that in the S0 group (4.63) (*p*< 0.05). The Fv/Fm in the S1, S2, and S3 groups were 0.86, 0.83, and 0.78, respectively, which decreased by 3.91%, 8.12%, and 9.46% compared with that in the S0 group (0.89) (*p*< 0.05). The qP in the S1, S2, and S3 groups were 0.96, 0.93, and 0.90, respectively, which decreased by 2.17%, 4.44%, and 6.82% compared with that in the S0 group (0.98) (*p*< 0.05). The ΦPSII in the S1, S2, and S3 groups were 0.69, 0.67, and 0.66, respectively, which decreased by 2.52%, 3.75%, and 6.14% compared with that in the S0 group (0.71) (*p*< 0.05). The ETR in the S1, S2, and S3 groups were 171.84, 160.83, and 152.80, respectively, which decreased by 4.45%, 11.12%, and 27.16% compared with that in the S0 group (180.17) (*p*< 0.05). The NPQ in the S1, S2, and S3 groups were 2.48, 2.89, and 3.36, respectively, which increased by 37.09%, 60.26%, and 82.78% compared with that in the S0 group (2.00) (*p*< 0.05).

### 3.2 Changes in spectral reflectance of rapeseed leaves under salinity stress

**Figure 5:**
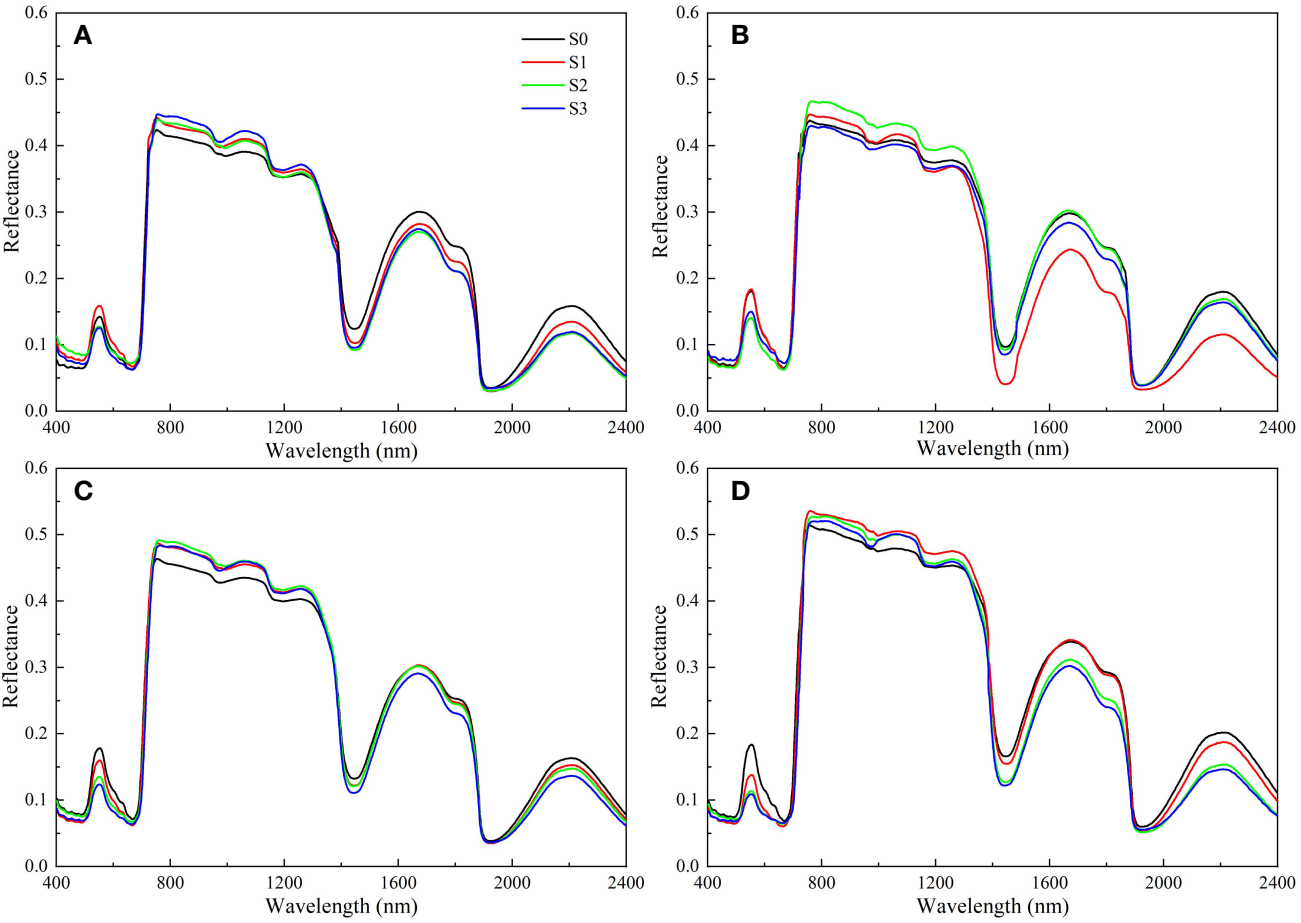
Changes in spectral reflectance of rapeseed leaves at different soil salinity levels. a, b, c, and d represent the spectral reflectance of salinity-stressed rapeseed leaves on day 10, 20, 30, and 40, respectively. S0, S1, S2, and S3 indicate that the content of NaCl in the soil is 0, 0.15, 0.3, and 0.45 g·kg^-1^, respectively.

The impacts of salinity treatments on rapeseed leaf spectral reflectance were similar, but the spectral reflectance of different groups were different (**Figure 5**). In the visible region (400-700 nm), the spectral reflectance decreased with the increase of soil salinity. On day 40, the peak at 553 nm for the S1, S2, and S3 groups decreased by 11.62%, 32.07%, and 44.19%, respectively compared with that for the S0 group. The spectral reflectance rose sharply in the range of 700 ~ 760 nm. In the near-infrared region, a high spectral reflectance was detected in 760 nm ~ 1100 nm, and the reflectance increased with the increase of soil salinity. The spectral reflectance curve for the S1, S2, and S3 groups did not show an obvious law in 1100 nm ~ 2400 nm with the increase of soil salinity, but gradually increased over time.

### 3.3 Construction of PLSR model based on the full band and different preprocessings

The SNV and CWT preprocessings improved the estimation accuracy of the PLSR model. With the increase of salinity stress time, the estimation accuracy first increased, peaked on day 30, and then decreased. The accuracy of the PLSR model in estimating CFPGE parameters were different under different spectral preprocessings. The Ci and Fv/F0 estimation accuracy of the PLSR model were the highest under CWT-3 preprocessing, with R^2^ of 0.758 and 0.523 on day 30, respectively. The Pn, Tr, ΦPSII, and ETR estimation accuracy of the PLSR model were the highest under CWT-4 preprocessing, with R^2^ of 0.654, 0.746, 0.752, and 0.746 on day 30, respectively. The Fv/Fm, qP, and NPQ estimation accuracy of the PLSR model were the highest under CWT-5 preprocessing, with R^2^ of 0.534, 0.519, and 0.633 on day 30, respectively. The gs estimation accuracy of the PLSR model was the highest under CWT-6 preprocessing, with R^2^ of 0.738 on day 30 (**Table 1**).

**Table 1:** Coefficient of determination (R^2^) of the PLSR models constructed based on different spectral preprocessing methods.

Stage	preprocessing	Pn	Tr	gs	Ci	ΦpsII	Fv/Fm	Fv/F0	qP	NPQ	ETR
10 d	R	0.248	0.275	0.299	0.422	0.288	0.289	0.249	0.254	0.267	0.274
	SNV	0.369	0.365	0.373	0.521	0.283	0.413	0.325	0.322	0.355	0.378
	CWT-1	0.274	0.321	0.324	0.513	0.315	0.363	0.275	0.306	0.362	0.486
	CWT-2	0.362	0.467	0.475	0.532	0.405	0.391	0.377	0.391	0.443	0.526
	CWT-3	0.377	0.511	0.477	**0.649**	0.424	0.484	**0.426**	0.437	0.503	0.537
	CWT-4	**0.456**	**0.613**	0.508	0.613	**0.508**	0.504	0.392	0.501	0.607	**0.608**
	CWT-5	0.448	0.463	0.545	0.517	0.405	**0.534**	0.302	**0.519**	**0.633**	0.435
	CWT-6	0.397	0.436	**0.594**	0.506	0.381	0.474	0.248	0.477	0.611	0.433
	CWT-7	0.331	0.338	0.322	0.406	0.314	0.234	0.233	0.438	0.547	0.334
	CWT-8	0.301	0.284	0.295	0.317	0.303	0.225	0.214	0.282	0.435	0.314
	CWT-9	0.207	0.225	0.294	0.276	0.256	0.215	0.206	0.277	0.292	0.277
	CWT-10	0.188	0.219	0.267	0.219	0.213	0.209	0.198	0.229	0.258	0.204
20 d	R	0.351	0.491	0.413	0.334	0.492	0.426	0.317	0.369	0.379	0.445
	SNV	0.489	0.538	0.627	0.555	0.568	0.556	0.423	0.407	0.459	0.582
	CWT-1	0.381	0.499	0.432	0.494	0.532	0.457	0.329	0.494	0.484	0.521
	CWT-2	0.446	0.553	0.498	0.553	0.607	0.462	0.485	0.508	0.485	0.548
	CWT-3	0.552	0.656	0.557	**0.691**	0.699	0.477	**0.504**	0.521	0.574	0.629
	CWT-4	**0.601**	**0.683**	0.662	0.682	**0.707**	0.567	0.499	0.589	0.671	**0.684**
	CWT-5	0.579	0.619	0.684	0.522	0.703	**0.648**	0.407	**0.621**	**0.698**	0.637
	CWT-6	0.491	0.514	**0.695**	0.503	0.61	0.431	0.396	0.541	0.627	0.456
	CWT-7	0.438	0.491	0.505	0.484	0.515	0.429	0.385	0.487	0.549	0.431
	CWT-8	0.366	0.489	0.464	0.403	0.489	0.418	0.309	0.444	0.464	0.423
	CWT-9	0.333	0.461	0.428	0.291	0.443	0.372	0.292	0.377	0.401	0.372
	CWT-10	0.305	0.399	0.386	0.25	0.368	0.332	0.272	0.292	0.359	0.248
30 d	R	0.371	0.504	0.495	0.433	0.508	0.565	0.343	0.399	0.422	0.463
	SNV	0.519	0.562	0.645	0.596	0.632	0.651	0.452	0.461	0.552	0.591
	CWT-1	0.432	0.568	0.442	0.444	0.555	0.598	0.369	0.516	0.496	0.541
	CWT-2	0.473	0.601	0.452	0.718	0.631	0.644	0.497	0.539	0.567	0.599
	CWT-3	0.554	0.699	0.622	**0.758**	0.692	0.709	**0.523**	0.561	0.631	0.732
	CWT-4	**0.654**	**0.746**	0.706	0.745	**0.752**	0.747	0.517	0.666	0.722	**0.746**
	CWT-5	0.609	0.716	0.713	0.546	0.706	**0.761**	0.482	**0.674**	**0.749**	0.656
	CWT-6	0.539	0.527	**0.738**	0.536	0.648	0.713	0.431	0.666	0.648	0.507
	CWT-7	0.499	0.502	0.537	0.497	0.577	0.612	0.402	0.556	0.61	0.485
	CWT-8	0.429	0.494	0.505	0.407	0.551	0.531	0.278	0.446	0.577	0.454
	CWT-9	0.411	0.465	0.485	0.318	0.494	0.452	0.218	0.396	0.442	0.402
	CWT-10	0.35	0.401	0.441	0.308	0.476	0.378	0.211	0.359	0.424	0.372
40 d	R	0.351	0.337	0.249	0.365	0.402	0.312	0.257	0.265	0.401	0.399
	SNV	0.381	0.422	0.289	0.46	0.493	0.354	0.279	0.343	0.497	0.495
	CWT-1	0.377	0.354	0.306	0.425	0.506	0.357	0.315	0.356	0.471	0.407
	CWT-2	0.402	0.517	0.338	0.517	0.581	0.481	0.422	0.368	0.541	0.533
	CWT-3	0.513	0.614	0.531	**0.612**	0.616	0.506	**0.509**	0.419	0.607	0.598
	CWT-4	**0.574**	**0.633**	0.657	0.575	**0.644**	0.561	0.497	0.434	0.669	**0.676**
	CWT-5	0.533	0.56	0.638	0.539	0.605	**0.607**	0.409	**0.523**	**0.682**	0.622
	CWT-6	0.516	0.509	**0.658**	0.529	0.523	0.555	0.399	0.426	0.624	0.439
	CWT-7	0.425	0.419	0.489	0.495	0.474	0.464	0.329	0.682	0.569	0.419
	CWT-8	0.354	0.409	0.318	0.336	0.432	0.425	0.293	0.377	0.541	0.412
	CWT-9	0.397	0.358	0.241	0.294	0.239	0.301	0.239	0.358	0.435	0.305
	CWT-10	0.217	0.313	0.236	0.265	0.221	0.251	0.236	0.214	0.443	0.313

R, raw spectral data; SNV, standard normal variate (SNV) transformation; CWT, continuous wavelet transformation. The decomposition scale of CWT was set as 2^1^ (CWT-1), 2^2^ (CWT-2),…, 2^10^ (CWT-10).

Bold values representing the optimal preprocessing corresponding R2 for different indicators.

### 3.4 Distribution of spectral features of photosynthetic gas exchange and chlorophyll fluorescence parameters

**Figure 6:**
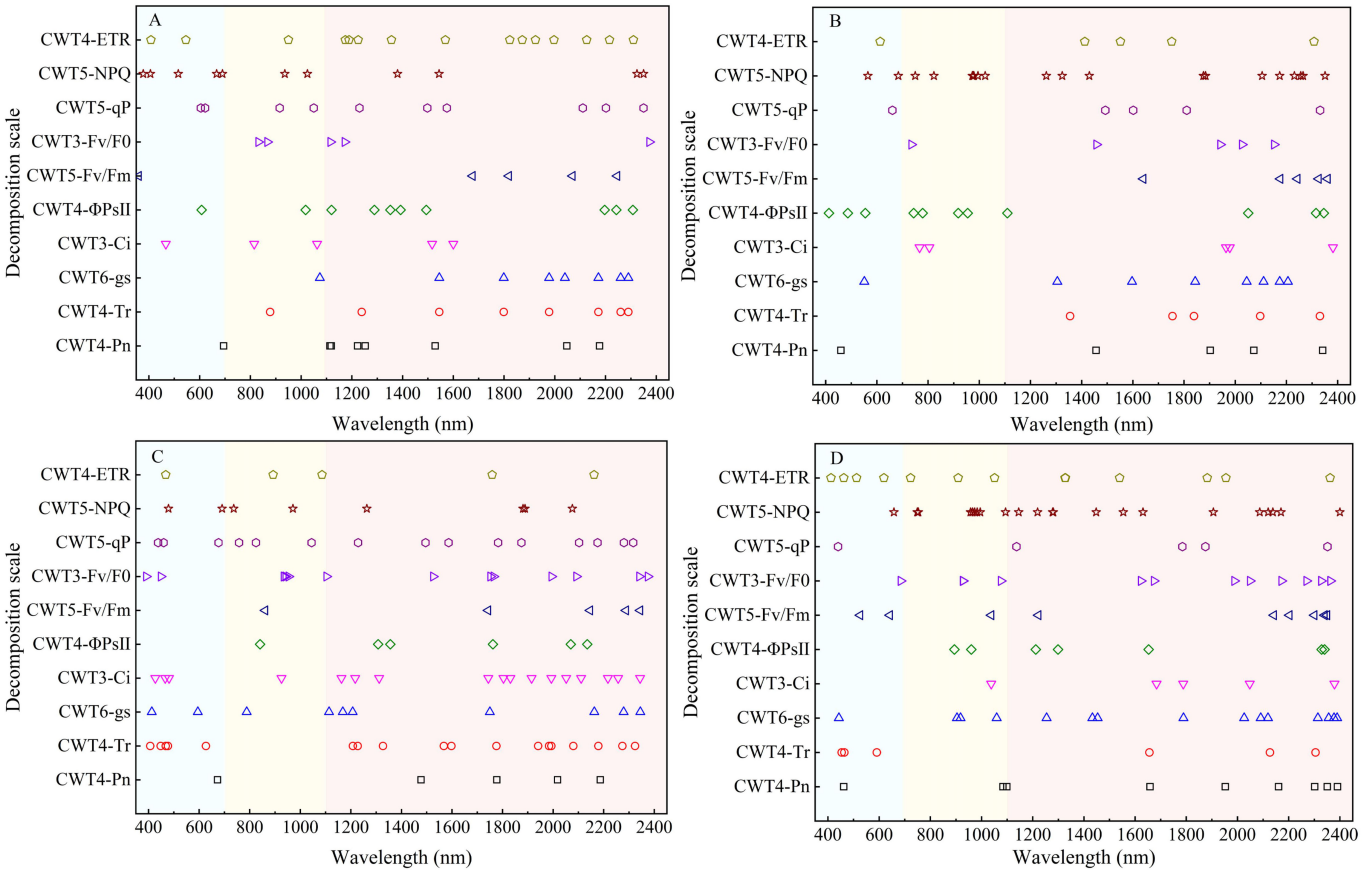
Distribution of spectral features of photosynthetic gas exchange and chlorophyll fluorescence parameters in rapeseed leaves at different growth stages.

The SPA was used for extracting spectral features to clarify distribution of the spectral features of CFPGE parameters. Most spectral features of the CFPGE parameters were in the red region (600 - 800 nm). In addition, some spectral features were in the blue-green region and the near-infrared region. The Fv/Fm and Fv/F0 had spectral features in 900 - 1000 nm, the Pn and gs had spectral features near 2100 nm, the NPQ, ETR, and Ci had spectral features in 1450 - 1650 nm, the qP had spectral features at 460 and 1019 nm, and the Tr and ΦPSII had spectral features around 482, 1453, 1600, and 2250 nm. The difference in the distribution of spectral features were not obvious between different periods (**Figure 6**).

### 3.5 Estimation accuracy of the PLSR and SVM models

**Figure 7:**
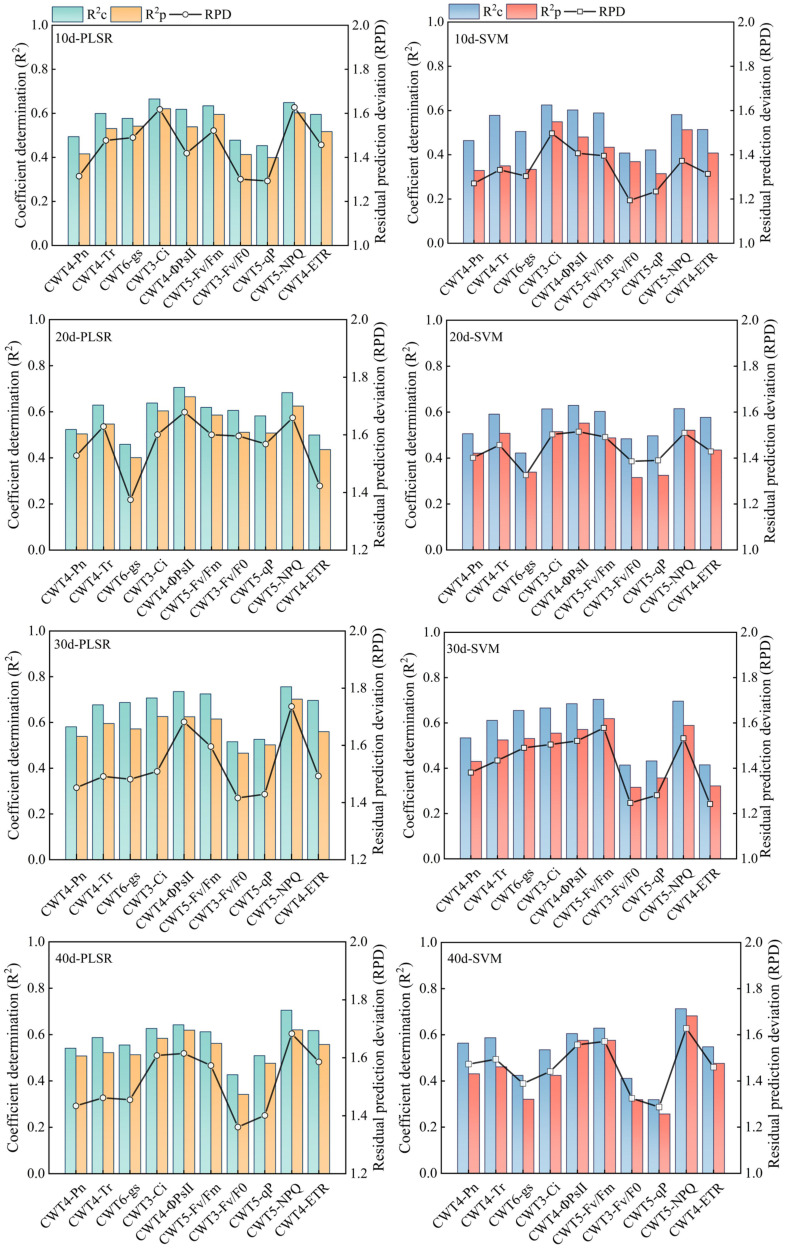
PLSR and SVM models constructed based on the spectral features extracted from the red region (600-800 nm).

**Figure 8:**
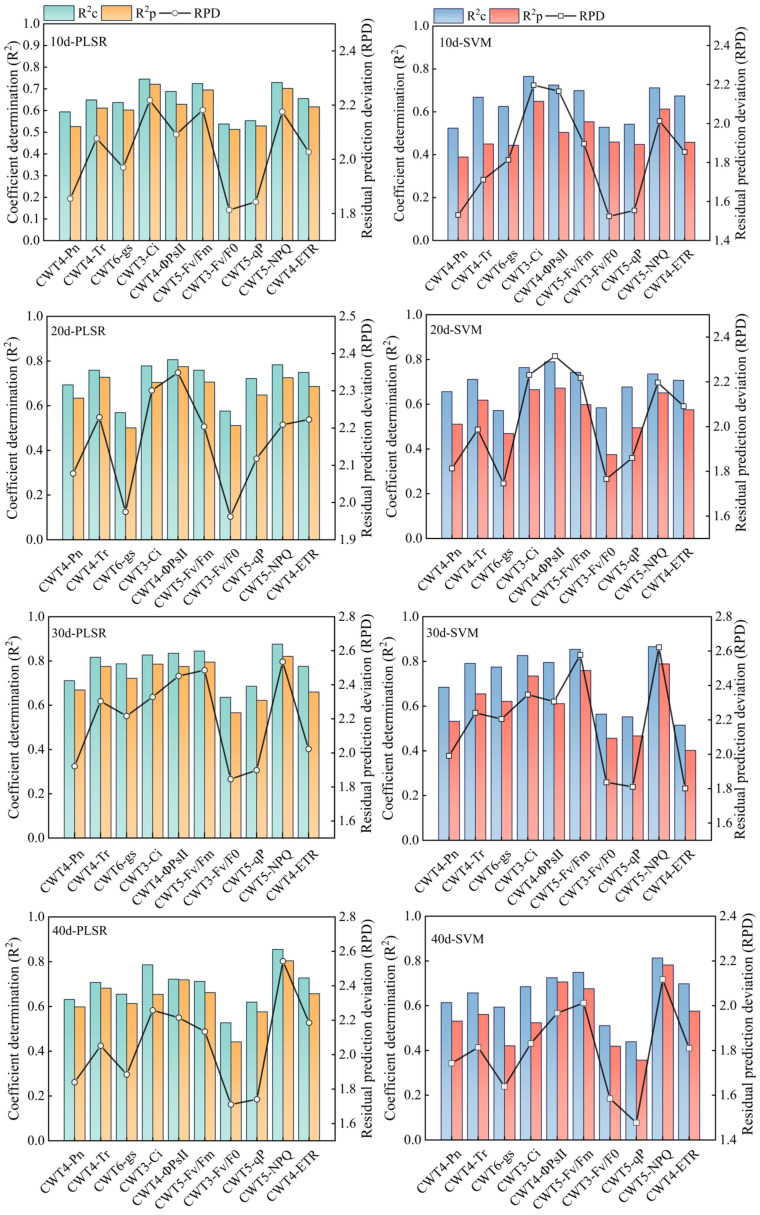
PLSR and SVM models constructed based on the spectral features extracted from the red, blue-green, and near-infrared regions (350 - 2500 nm).

PLSR and SVM models were constructed based on the spectral features extracted from the red region (RSF) and the spectral features extracted from the red, blue-green, and near-infrared regions (FSF) (**Figures 7**, **8**). The accuracy of the PLSR and SVM models constructed based on the RSF were generally lower than that of the models based on the FSF. Taking the PLSR and SVM models constructed based on the FSF as an example (**Figure 8**), with the increase of salinity stress time, the accuracy of the estimation models were different, and the accuracy first increased and then decreased (peaked on day 30). However, the accuracy of the PLSR model was higher than that of the SVM model. On day 10, the CWT3- PLSR model had the highest Ci estimation accuracy, with R^2^c of 0.752, R^2^p of 0.713, and RPD of 2.04. On day 20, the CWT4-PLSR model had the highest ΦPSII estimation accuracy, with R^2^c of 0.817, R^2^p of 0.786, and RPD of 2.36. On day 30, the CWT5-PLSR model had the highest Fv/Fm estimation accuracy, with R^2^c of 0.886, R^2^p of 0.815, and RPD of 2.58. On day 40, the CWT5-PLSR model had the highest NPQ estimation accuracy, with R^2^c of 0.857, R^2^p of 0.803, and RPD of 2.57.

### 3.6 Model validation

**Figure 9:**
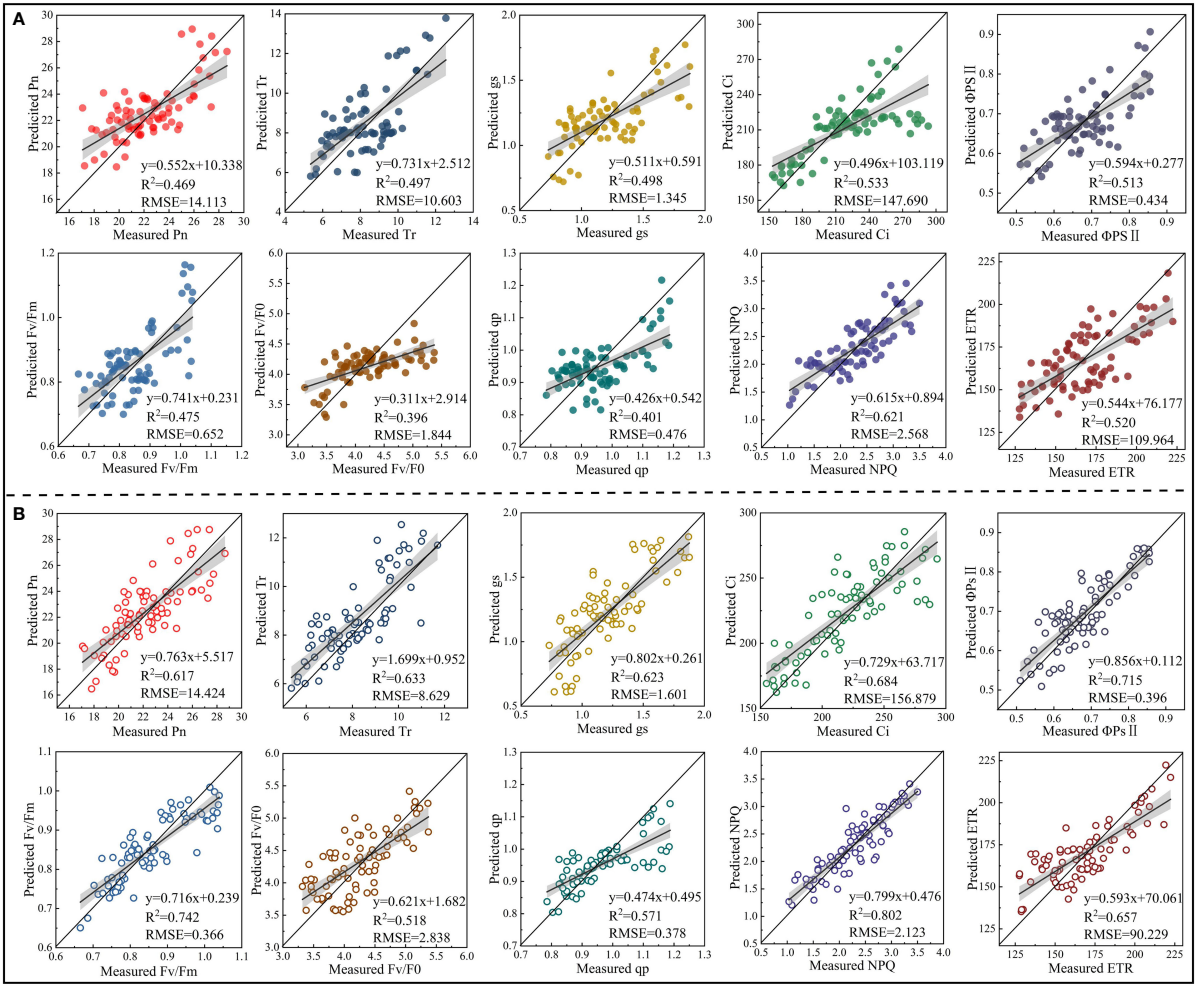
Model validation (n = 80). **(A)** is the validation results of the accuracy of the model constructed based on the spectral features extracted from the red region in estimating photosynthetic gas exchange and chlorophyll fluorescence parameters of rapeseed leaves under 30-day salinity stress. **(B)** is the validation results of the accuracy of the model constructed based on the spectral features extracted from red, blue-green, and near-infrared regions in estimating photosynthetic gas exchange and chlorophyll fluorescence parameters of rapeseed leaves under 30-day salinity stress.

To verify the universality and stability of the models constructed, the PLSR models constructed based on the RSF and FSF were tested by the validation set. The PLSR models constructed based on the RSF and FSF with the highest accuracy on day 30 were analyzed as examples, and the validation results for other periods are shown in **Figures S2-S4**. The validation results showed that the model based on the RSF generally had a low accuracy in estimating CFPGE parameters. Except for the R^2^ of the model in estimating NPQ (R^2^: 0.621, RMSE: 2.568), the R^2^ of the model in estimating other parameters were lower than 0.6 (**Figure 9A**). The accuracy of the model based on the FSF was higher than that of the model based on the RSF. The accuracy of the FSF-PLSR model was low in estimating Fv/F0 (R^2^: 0.518, RMSE: 2.838) and qp (R^2^: 0.571 and RMSE, RMSE: 0.378). The R^2^ of the FSF-PLSR model were higher than 0.6 in estimating other parameters, among which the accuracy of the model was the highest in estimating NPQ, with R^2^ of 0.802 and RMSE of 2.131 (**Figure 9B**).

## 4 Discussion

### 4.1 Responses of chlorophyll fluorescence and photosynthetic gas exchange parameters of rapeseed leaves to salinity stress

Salinity stress has multifaceted effects on crop growth and metabolism, especially photosynthesis (Ben-asher et al., 2006). Studies have shown that the effects of salinity stress on crop photosynthesis mainly include stomatal and non-stomatal limitations (Ouerghi et al., 2000). The gs of plants leaves decreases under salinity stress, if Ci decreases, the main factor leading to the decrease in photosynthetic rate is stomatal limitation; If Ci increases or does not change, the main factor leading to the decrease in photosynthetic rate is nonstomatal limitation (Farquhar and Sharkey, 1982; Yeison et al., 2022). This study found that with the increase of soil salinity, the gas exchange parameters Pn, gs, and Tr of rapeseed leaves decreased, and Ci increased. This indicates that non-stomatal limitation is the main factor leading to the decrease in photosynthetic rate in this study (Farquhar and Sharkey, 1982). This is consistent with the results of Zhang et al. (2017). This is mainly due to that salinity stress leads to a large accumulation of salt ions in cells, destroying chloroplast structure. The reduction of CO_2_ in the mesophyll cell wall of chloroplasts is blocked, and the activity of ribulose-bisphosphate carboxylase is reduced, resulting in damage to leaf photosynthetic organs and decreased photosynthetic activity of the mesophyll cells (Parida et al., 2004; Ge et al., 2007; Sudhir and Murthy, 2004).

The decrease in photosynthetic rate will inevitably affect the absorption and transformation of light energy by crops, especially the photochemical activity (Foyer and Noctor, 2000). Studies have shown that the primary damaged photosynthesis organ is closely related to the potential activity of PSII. The photosystem is easily damaged by high salinity, resulting in decreases in Fv/Fm and Fv/Fo (Sun et al., 2012). In this study, the NPQ of rapeseed leaves showed an increasing trend, while other fluorescence parameters decreased with the increase of soil salinity. This indicates that salinity stress leads to photoinhibition in leaves (Xu et al., 1999), and the light energy used for photochemical reactions decreases. The results are consistent with those of Liu’s study on ryegrass (*Lolium perenne*) (Liu et al., 2012). This is mainly due to that under salinity stress, the proportion of light energy captured by rapeseed leaves for photochemical reactions decreases, and the proportion of light energy for heat dissipation increases. This inhibits the potential activity of PSII reaction center, and affects the excitation energy distribution of PSII of rapeseed leaves. The plants adapt to the salinity stress environment by increasing the excitation energy consumed by heat dissipation (Hendrickson et al., 2004).

### 4.2 Distribution of spectral features of photosynthetic gas exchange and chlorophyll fluorescence parameters of rapeseed leaves

The CFPGE parameters are important for evaluating crop photosynthesis under salinity stress. Due to these parameter changes lead to the changes in spectral reflectance, the mechanism by which spectral reflectance responding to leaf photosynthesis can be determined (Liu et al., 2013; Dechant et al., 2017). At present, there is still controversy over the distribution of spectral features of the CFPGE parameters. This is due to the fact that except for the red region, the CFPGE signals in the blue-green and near-infrared regions are weak and cannot be detected by hyperspectral remote sensing (Wen et al., 2022). Therefore, most scholars believed that the spectral features of crop leaf CFPGE parameters were concentrated in the red region, and constructed vegetation indices based on the red region to predict the CFPGE parameters (Buschmann et al., 2000; Zarco-Tejada et al., 2000).

In this study, the SNV and CWT were used to preprocess the raw spectra. It was found that the spectral preprocessing by CWT was better. This may be due to that the CWT can effectively remove noises in the spectrum, enhance the relationship between spectra and CFPGE parameters, thereby improving the prediction accuracy and stability (Zhang et al., 2020). Zhao et al. (2021) also demonstrated the potential of CWT in fluorescence signal extraction as a means to rapidly detect crop leaf CFPGE. In this study, the spectral features of CFPGE parameters were extracted by the SPA. The results showed that most of the spectral features of CFPGE parameters were distributed in the red region. This is consistent with previous findings. For example, Tan et al. (2012) reported that the spectral features of Fv/Fm of maize were at 445, 680, and 800 nm. Zhang et al. (2012) reported that the spectral features of Fv/Fm and qP of *Suaeda glauca* were near 680 and 935 nm. Magney et al. (2014) reported that spectral bands of 531 and 570 nm had the greatest correlation with the NPQ of winter wheat, and changes in NPQ could be monitored using (R531-R570)/(R531+ R570).However, in this study, it was also found that some spectral features of CFPGE parameters were in the blue-green and near-infrared regions. For example, NPQ, ETR, and Ci had multiple absorption peaks at 1450-1650 nm. This may be due to the fact that -NH3^+^ in amino acid molecules has a strong symmetric band in this region (Zhang, 2009). The Fv/Fm had a peak at 987 nm. This reflects the C-H bond of rapeseed leaf fat. The qP had a peak at 1019 nm. This spectral feature is an N-H bond reflecting rapeseed leaf protein (Zhao et al., 2021). The Tr and ΦPSII had spectral features at 482, 578, 1453, 1600, and 2250 nm in the shortwave near-infrared region, while the bands at 1453 and 1600 nm could indicate the high water content of crop leaves (Wang et al., 2001). It can be seen that CFPGE parameters are not only closely related to crop leaf pigments, but also sensitive to changes in leaf biochemical components (e.g., protein, amino acid, and water content) and leaf internal structure (Meroni et al., 2009; Zarco-Tejada et al., 2009). This study confirmed that CFPGE parameters had spectral features not only in the red region, but also in the blue-green and near-infrared regions. This findings can further improve the estimation accuracy based on the spectral features in 600-800 nm in previous studies (Zheng et al., 2021).

### 4.3 Effect of different modeling strategies on the estimation accuracy of chlorophyll fluorescence and photosynthetic gas exchange parameters

Spectral feature extraction and modeling strategy have a great impact on the accuracy of spectral estimation of crop growth parameters (Li et al., 2014). There have been many studies on the estimation of crop CFPGE parameters using the spectral features in the red region (600-800 nm), but whether the blue-green (350-600 nm) and near-infrared (800-2500 nm) regions have potential for estimating crop CFPGE parameters needs to be further explored (Buschmann et al., 2000). In this study, it was found that CFPGE parameters had spectral features not only in the red region, but also in the blue-green and near-infrared regions. To explore the influence of spectral features of different regions on the spectral estimation of crop CFPGE parameters, this study constructed CFPGE parameter estimation models based on the full spectra, spectral features in the red region, and spectral features in the red, blue-green, and near-infrared regions, respectively. The results showed that the accuracy of the PLSR and SVM models constructed based on the spectral features was higher than that of the model based on full spectra. This may be due to that the massive spectral data contain some redundant and collinear data that may negatively impact the accuracy and universality of the estimation model. Therefore, eliminating the redundant information by extracting spectral features from the full spectra is conducive to improving the accuracy and stability of the estimation model (Araujo et al., 2001; Mario et al., 2019). In this study, the accuracy of the PLSR models were higher than that of the SVM models. This may be due to that PLSR could remove redundant information and noises, and effectively solve the problems of overfitting and multicollinearity (Li et al., 2014). It is worth noting that the accuracy of the CFPGE parameter estimation model constructed based on the spectral features in the red, blue-green, and near-infrared regions was significantly higher than that of red region. This may be due to the fact that the model constructed based on the RSF is susceptible to the influence of leaf pigments, biochemical components, and moisture, while most of the spectral features of these factors are distributed in the blue-green and near-infrared regions. Therefore, adding the spectral features of blue-green and near-infrared regions on the basis of the RSF could effectively reduce the interference of the above factors, and improve the accuracy and stability of the model (Hansen et al., 2003; Weber et al., 2012). In this study, the estimation models constructed were further validated (**Figure 9**). It was found that the accuracy of the estimation model based on the spectral features in the red, blue-green, and near-infrared regions was higher than that of the red region. This verifies that the fusion of spectral features of blue-green and near-infrared regions can improve the stability and universality of the CFPGE parameter estimation model. However, it still needs to be widely verified in different experimental environments with different crops, and the reliability of this method also needs to be further studied.

## 5 Conclusion

To quickly and non-destructively monitor the photosynthetic performance of rapeseed leaves under salinity stress, in this study, the effects of salinity stress on rapeseed leaf photosynthesis were explored, and the spectral data of rapeseed leaves were acquired. After preprocessing the spectral data using the CWT, the spectral features of rapeseed leaf CFPGE parameters in the blue-green, red, and near-infrared regions under salinity stress were extracted by SPA. Finally, CFPGE parameter estimation models based on PLSR and SVM were constructed.

Under salinity stress, the gas exchange parameters Pn, gs, and Tr and the chlorophyll fluorescence parameters Fv/F0, Fv/Fm, qP, ΦPSII, and ETR decreased, while the Ci and NPQ increased. After CWT preprocessing and the extraction of spectral features of rapeseed leaf CFPGE parameters using SPA, it was found that the spectral features of rapeseed leaf CFPGE parameters were not only distributed in the red region, but also in the blue-green and near-infrared regions. The accuracy of the CFPGE parameter estimation model constructed based on the spectral features in the red region had the highest Fv/Fm estimation accuracy at 30 d, and the R^2^c, R^2^p, and RPD were 0.723, 0.585, and 1.68, respectively. On this basis, the spectral features in the red, blue-green, and near-infrared regions were fused to construct an estimation model, and the R^2^c, R^2^p, and RPD reached 0.886, 0.815, and 2.58, respectively. Therefore, the fusion of spectral features in the red, blue-green, and near-infrared regions significantly improved the model accuracy. This study provides a technical reference for the accurate spectral estimation of crop leaf CFPGE parameters, and help us better understand the light absorption and protection of photosynthetic system of crops under environmental stress.

## Data availability statement

The original contributions presented in the study are included in the article/**Supplementary Material**. Further inquiries can be directed to the corresponding authors.

## Author contributions

JW: Conceptualization, Data curation, Investigation, Methodology, Software, Writing – original draft, Writing – review & editing. TT: Methodology, Software, Writing – review & editing. HW: Conceptualization, Funding acquisition, Resources, Writing – original draft, Writing – review & editing. JC: Conceptualization, Formal Analysis, Methodology, Writing – original draft, Writing – review & editing. XS: Formal Analysis, Software, Writing – original draft. JS: Data curation, Software, Validation, Writing – original draft. TL: Software, Validation, Writing – original draft. WL: Data curation, Formal Analysis, Writing – original draft. MZ: Data curation, Formal Analysis, Writing – original draft. WZ: Data curation, Formal Analysis, Software, Writing – original draft.

## References

[B1] Araújo M. C. U. Saldanha T. C. B. Galvo R. K. H. Yoneyama T. Visani V. (2001). The successive projections algorithm for variable selection in spectroscopic multicomponent analysis. Chemometrics Intelligent Lab. Syst. 57, 65–73. doi: 10.1016/S0169-7439(01)00119-8

[B2] Ashourloo D. Mobasheri M. R. Huete A. (2014). Developing two spectral disease indices for detection of wheat leaf rust (Puccinia triticina). Remote Sens. 6, 4723–4740. doi: 10.3390/rs6064723

[B3] Baker N. R. Rosenqvist E. (2004). Applications of chlorophyll fluorescence can improve crop production strategies: An examination of future possibilities. J. Exp. Botany 55, 1607–1621. doi: 10.1093/jxb/erz535 15258166

[B4] Banakar M. H. Amiri H. Ardakani M. R. S. Ranjbar G. H. (2022). Susceptibility and tolerance of fenugreek (Trigonella foenum-graceum L.) to salinity stress: Physiological and biochemical inspections. Environ. Exp. Botany 194, 104748. doi: 10.1016/j.envexpbot.2021.104748

[B5] Bao S. D. (2000). Soil and Agricultural Chemistry Analysis. 3rd edn (Beijing: China Agriculture Press).

[B6] Barnes R. J. Dhanoa M. S. Lister J. (1989). Standard normal variate transformation and de-trending of near-infrared diffuse reflectance spectra. Appl. Spectrosc. 43, 772–777. doi: 10.1366/0003702894202201

[B7] Ben-asher J. Tsuyuki I. Bravdo B. A. Sagi M. (2006). Irrigation of grapevines with saline water: I. Leaf area index, stomatal conductance, transpiration and photosynthesis. Agric. Water Manag. 83, 13–21. doi: 10.1016/j.agwat.2006.01.002

[B8] Buschmann C. Langsdorf G. Lichtenthaler H. K. (2000). Imaging of the blue, green, and red fluorescence emission of plants: an overview. Photosynthetica 38, 483–491. doi: 10.1023/A:1012440903014

[B9] Chen J. Yao X. Huang F. Liu Y. Yu Q. Wang N.. (2016). N status monitoring model in winter wheat based on image processing. Trans. Chin. Soc. Agric. Engineering 32, 163–170. doi: 10.11975/j.issn.1002-6819.2016.04.023

[B10] Cheng T. Rivard B. Sanchez-azofeifa A. (2010). Spectroscopic determination of leaf water content using continuous wavelet analysis. Remote Sens. Environ. 115, 659–670. doi: 10.1016/j.rse.2010.11.001

[B11] Clarke K. R. Green R. H. (1988). Statistical design and analysis for a ‘biological effects’ study. Mar. Biol. 46, 213–226. doi: 10.3354/meps046213

[B12] Dechant B. Cuntz M. Vohland M. Schulz E. Doktor D. (2017). Estimation of photosynthesis traits from leaf reflectance spectra: Correlation to nitrogen content as the dominant mechanism. Remote Sens. Environ. 196, 279–292. doi: 10.1016/j.rse.2017.05.019

[B13] Fariduddin H. Q. (2013). Salt-induced modulation in growth, photosynthesis and antioxidant system in two varieties of Brassica juncea. Saudi J. Biol. Sci. 20, 183–193. doi: 10.1016/j.sjbs.2013.01.006 23961235 PMC3730539

[B14] Farquhar G. D. Sharkey T. D. (1982). Stomatal conductance and photosynthesis. Annu. Rev. Plant Physiol. 33, 317–345. doi: 10.1146/annurev.pp.33.060182.001533

[B15] Feng L. Zhu S. Zhang C. Bao Y. D. Feng X. P. He Y. (2018). Identification of maize kernel vigor under different accelerated aging times using hyperspectral imaging. Molecules 23, 3078. doi: 10.3390/molecules23123078 30477266 PMC6321087

[B16] Foyer C. H. Noctor G. (2000). Tansley review No. 112. Oxygen processing in photosynthesis: Regulation and signalling. New Phytologist. 146, 359–388. doi: 10.1046/j.1469-8137.2000.00667.x

[B17] Galvo R. K. H. Araújo M. C. U. Fragoso W. D. Silva E. C. Paiva H. M. (2008). A variable elimination method to improve the parsimony of mlr models using the successive projections algorithm. Chemometrics Intelligent Lab. Syst. 92, 83–91. doi: 10.1016/j.chemolab.2007.12.004

[B18] Ge J. L. Jiang C. D. Shi L. Gu W. B. Zhang J. Z. Ren D. M. (2007). Effect of slight salt-stress on excitation energy distribution of photosynthesis in sweet sorghum. J. Shenyang Agric. Univ. 38, 366–369.

[B19] Grisanti E. Totska M. Huber S. Calderon C. K. Otto M. (2018). Dynamic localized snv, peak snv, and partial peak snv: novel standardization methods for preprocessing of spectroscopic data used in predictive modeling. J. Spectrosc. 2018, 1–14. doi: 10.1155/2018/5037572

[B20] Hamzeh S. Naseri A. A. Alavipanah S. K. Mojaradi B. Bartholomeus H. M. Clevers J. G. P. W.. (2013). Estimating salinity stress in sugarcane fields with spaceborne hyperspectral vegetation indices. Int. J. Appl. Earth Observ. Geoinform. 21, 282–290. doi: 10.1016/j.jag.2012.07.002

[B21] Hansen P. M. Jorgensen J. R. Thomsen A. (2003). Predicting grain yield and protein content in winter wheat and spring barley using repeated canopy reflectance measurements and partial least squares regression. J. Agric. Sci. 139, 307–318. doi: 10.1017/S0021859602002320

[B22] Hendrickson L. Furbank R. T. Chow W. S. (2004). A simple alternative approach to assessing the fate of absorbed light energy using chlorophyll fluorescence. Photosynth. Res. 82, 73–81. doi: 10.1023/B:PRES.0000040446.87305.f4 16228614

[B23] Hniličková H. Hnilička F. Martinková J. Kraus K. (2017). Effects of salinity stress on water status, photosynthesis and chlorophyll fluorescence of rocket. Plant Soil Environ. 64, 362–367. doi: 10.17221/398/2017-PSE

[B24] Hong Y. S. Liu Y. L. Chen Y. Y. Liu Y. F. Yu L. Liu Y.. (2019). Application of fractional-order derivative in the quantitative estimation of soil organic matter content through visible and near-infrared spectroscopy. Geoderma 337, 758–769. doi: 10.1016/j.geoderma.2018.10.025

[B25] Huang W. J. Shi Y. Dong Y. Y. Ye H. C. Wu M. Q. Cui B.. (2019). Progress and prospects of crop diseases and pests monitoring by remote sensing. Smart Agric. 1, 1–11. doi: 10.12133/j.smartag.2019.1.4.201905-SA005

[B26] Inoue Y. Guérif M. Baret F. Skidmore A. Gitelson A. Schlerf M.. (2016). Simple and robust methods for remote sensing of canopy chlorophyll content: A comparative analysis of hyperspectral data for different types of vegetation. Plant Cell Environ. 39, 2609–2623. doi: 10.1111/pce.12815 27650474

[B27] Jia M. Li W. Wang K. K. Zhou C. Yao X. Cheng T.. (2019). A newly developed method to extract the optimal hyperspectral feature for monitoring leaf biomass in wheat. Comput. Electron. Agric. 165, 104942. doi: 10.1016/j.compag.2019.104942

[B28] Koger C. H. Bruce L. M. Shaw D. R. Reddy K. N. (2003). Wavelet analysis of hyperspectral reflectance data for detecting pitted morningglory (Ipomoea lacunosa) in soybean (Glycine max). Remote Sens. Environ. 86, 108–119. doi: 10.1016/S0034-4257(03)00071-3

[B31] Li B. (2021). Development of PSII potential maximum photosynthetic capacity detector for protected crops based on visible-near infrared technology. Northwest A&F Univ. doi: 10.27409/d.cnki.gxbnu.2021.001576

[B29] Li F. Mistele B. Hu Y. C. Chen X. P. Schmidhalter U. (2014). Reflectance estimation of canopy nitrogen content in winter wheat using optimised hyperspectral spectral indices and partial least squares regression. Eur. J. Agronomy. 52, 198–209. doi: 10.1016/j.eja.2013.09.006

[B30] Li C. Zhao T. L. Li C. Mei L. Yu E. Dong Y. T.. (2017). Determination of gossypol content in cottonseeds by near infrared spectroscopy based on Monte Carlo uninformative variable elimination and nonlinear calibration methods. Food Chem. 221, 990–996. doi: 10.1016/j.foodchem.2016.11.064 27979304

[B32] Liu J. X. Wang X. Wang R. J. Jia H. Y. (2012). Photosynthetic physiological response of Lolium perenne to NaHCO^3^ stress. Acta Prataculturae Sinica 21, 184–190.

[B34] Liu N. Xing Z. Z. Zhao R. M. Qiao L. Li M. Z. Liu G.. (2020). Analysis of chlorophyll concentration in potato crop by coupling continuous wavelet transform and spectral variable optimization. Remote Sens. 12, 2826. doi: 10.3390/rs12172826

[B33] Liu L. Y. Zhang Y. J. Jiao Q. J. Peng D. L. (2013). Assessing photosynthetic light-use efficiency using a solar-induced chlorophyll fluorescence and photochemical reflectance index. Int. J. Remote Sens. 34, 4264–4280. doi: 10.1080/01431161.2013.775533

[B35] Luo J. X. (1985). Improvement of Saline Alkaline Land in Xinjiang Reclamation Area (Beijing: Water Resources and Electric Power Press).

[B36] Magney T. S. Eusden S. A. Eitel J. U. H. Logan B. A. Jiang J. Vierling L. A. (2014). Assessing leaf photoprotective mechanisms using terrestrial LiDAR: towards mapping canopy photosynthetic performance in three dimensions. New Phytologist. 201, 344–356. doi: 10.1111/nph.12453 24032717

[B37] Mahanti N. K. Chakraborty S. K. Kotwaliwale N. Vishwakarma A. K. (2020). Chemometric strategies for nondestructive and rapid assessment of nitrate content in harvested spinach using vikmIR spectroscopy. J. Food Sci. 85, 3653–3662. doi: 10.1111/1750-3841.15420 32888324

[B1002] Mario H. J. Paoletti M. E. Plaza J. Plaza A. Li J. (2019). Hyperspectral image classification using random occlusion data augmentation. IEEE Geosci. Remote Sens. Lett. 16, 1751–1755. doi: 10.1109/LGRS.2019.2909495

[B38] Meroni M. Rossini M. Guanter L. Alonso L. Rascher U. Colombo R.. (2009). Remote sensing of solar-induced chlorophyll fluorescence: review of methods and applications. Remote Sens. Environ. 113, 2037–2051. doi: 10.1016/j.rse.2009.05.003

[B39] Mohammed G. H. Colombo R. Middleton E. M. Rascher U. Zarco-Tejada P. J. (2019). Remote sensing of solar-induced chlorophyll fluorescence (SIF) in vegetation: 50 years of progress. Remote Sens. Environ. 231, 111177. doi: 10.1016/j.rse.2019.04.030 33414568 PMC7787158

[B40] Ouerghi Z. Conrnic G. Roudani M. (2000). Effect of NaCl on photosynthesis of two wheat species (Triticum durum and T. aestivum) differing in their sensitivity to salt stress. Plant Physiol. 156, 335–340. doi: 10.1016/S0176-1617(00)80071-1

[B41] Parida A. K. Das A. B. Mohanty P. (2004). Investigations on the antioxidative defense responses to NaCl stress in a mangrove, Bruguiera parviflora: differential regulations of isoforms of some antioxidative enzymes. Plant Growth Regul. 42, 213–226. doi: 10.1023/B:GROW.0000026508.63288.39 15202709

[B42] Porcar-Castell A. Tyystjärvi E. Atherton J. van der Tol C. Flexas J. Pfündel E. E.. (2014). Linking chlo-rophyll a fluorescence to photosynthesis for remote sensing applications: mechanisms and challenges. J. Exp. Botany 65, 4065–4095. doi: 10.1093/jxb/eru191 24868038

[B43] Qadir M. Quillérou E. Nangia V. Murtaza G. Singh M. Thomas R. J.. (2014). Economics of salt-in-duced land degradation and restoration. Natural Resour. Forum 38, 282–295. doi: 10.1111/1477-8947.12054

[B44] Sudhir P. Murthy S. D. S. (2004). Effects of salt stress on basic processes of photosynthesis. Photosynthetica 42, 481–486. doi: 10.1007/S11099-005-0001-6

[B45] Sun L. Zhou Y. F. Li F. X. Xiao M. J. Tao Y. Xu W. J.. (2012). Impacts of salinity stress on characteristics of photosynthesis and chlorophyll fluorescence of sorghum seedlings. Scientia Agric. Sinica 45, 3265–3272. doi: 10.3864/j.issn.0578-1752.2012.16.005

[B46] Tan C. W. Huang W. J. Jin X. L. Wang J. C. Tong L. Wang J. H.. (2012). Using hyperspectral vegetation index to monitor the chlorophyll fluorescence parameters Fv/Fm of compact corn. Spectrosc. Spectral Anal. 32, 1287–1291. doi: 10.3964/j.issn.1000-0593(2012)05-1287-05 22827074

[B47] Tian T. Wang J. G. Wang. H. J. Cui J. Shi X. Y. Song J. H.. (2022). Synergistic use of spectral features of leaf nitrogen and physiological indices improves the estimation accuracy of nitrogen concentration in rapeseed. Int. J. Remote Sens. 43, 2755–2776. doi: 10.1080/01431161.2022.2068359

[B48] Tirado S. B. Dennis S. S. Enders T. A. Springer N. M. (2020). Utilizing top-down hyperspectral imaging for monitoring genotype and growth conditions in maize. Cold Spring Harbor Laboratory 23. doi: 10.1101/2020.01.21.914069

[B50] Wang J. Z. Ding J. L. Yu D. L. Teng D. X. He B. Chen X. Y.. (2020). Machine learning-based detection of soil salinity in an arid desert region, Northwest China: A comparison between Landsat-8 OLI and Sentinel 2 MSI. Sci. Total Environ. 707, 136092. doi: 10.1016/j.scitotenv.2019.136092 31972911

[B49] Wang J. H. Zhao C. J. Guo X. W. Tian Q. J. (2001). Study on the water status of wheat leaves diagnosed by the spectral reflectance. Sci. Agric. Sinica 1, 104–107.

[B51] Weber V. S. Araus J. L. Cairns J. E. Sanchez C. Melchinger A. E. Orsini E. (2012). Prediction of grain yield using reflectance spectra of canopy and leaves in maize plants grown under different water regimes. Field Crops Res. 128, 82–90. doi: 10.1016/j.fcr.2011.12.016

[B52] Wen S. Y. Shi N. Lu J. W. Gao Q. W. Hu W. R. Cao Z. D. Y.. (2022). Continuous wavelet transform and back propagation neural network for condition monitoring chlorophyll fluorescence parameters Fv/Fm of rice leaves. Agriculture 12, 1197–1197. doi: 10.3390/AGRICULTURE12081197

[B53] Wu S. G. Yu X. J. Li K. Jiang Y. J. Zhang R. M. (2014). Effects of sea salt on the reflectance spectra and chlorophyll fluorescence parameters of green bamboo leaves. Acta Ecol. Sinica 34, 4920–4930. doi: 10.5846/stxb201309232337

[B54] Xu C. C. Li D. Q. Zou Q. Zhang J. H. (1999). Effect of drought on chlorophyll fluorescence and xanthophyll cycle components in winter wheat leaves with different ages. Acta Phytophysiol. Sinica 25, 29–37. doi: 10.3321/j.issn:1671-3877.1999.01.005

[B55] Xue H. Y. Zhang Y. J. Liu L. T. Sun H. C. Li C. D. (2013). Effects of drought stress and rewatering on cotton leaf spectrum, photosynthesis and fluorescence parameters. Scientia Agric. Sinica 46, 2386–2393. doi: 10.3864/j.issn.0578-1752.2013.11.024

[B1001] Yao X. Si H. Y. Cheng T. Jia M. Chen Q. Tian Y. C.. (2018). Hyperspectral estimation of canopy leaf biomass phenotype per ground area using a continuous wavelet analysis in wheat. Front. Plant Sci. 9, 1360. doi: 10.3389/fpls.2018.01360 30319667 PMC6167447

[B56] Yeison M. Q. Liz P. M. Eduardo B. (2022). Predictive models of drought tolerance indices based on physiological, morphological and biochemical markers for the selection of cotton ( gossypium hirsutum l.) varieties. J. Integr. Agric. 21, 1310–1320. doi: 10.1016/S2095-3119(20)63596-1

[B59] Zarco-Tejada P. J. Berni J. A. J. Suárez L. Sepulcre-Cantó G. Morales F. Miller J. R. (2009). Imaging chlorophyll fluorescence with an airborne narrow-band multispectral camera for vegetation stress detection. Remote Sens. Environ. 113, 1262–1275. doi: 10.1016/j.rse.2009.02.016

[B57] Zarco-Tejada P. J. Miller J. R. Mohammed G. H. Noland T. L. Sampson P. H. (2000). Chlorophyll fluorescence effects on vegetation apparent reflectance: II. Laboratory and airborne canopy-level measurements with hyperspectral data. Remote Sens. Environ. 74, 596–608. doi: 10.1016/S0034-4257(00)00149-8

[B58] Zarco-Tejada P. J. Pushnik J. C. Dobrowski S. Z. Ustin S. L. (2003). Steady state chlorophyll a fluorescence detection from canopy derivative reflectance and double-peak red-edge effects. Remote Sens. Environ. 84, 283–294. doi: 10.1016/S0034-4257(02)00113-X

[B60] Zhang Z. X. (2009). Organic Spectral Analysis (Beijing: People's Medical Publishing Press).

[B65] Zhang S. Y. Fei T. Chen Y. Y. Yang J. X. Qu R. Xu J.. (2023). Identifying cadmium and lead Co-accumulation from living rice blade spectrum. Environ. Pollut. 338, 122618. doi: 10.1016/j.envpol.2023.122618 37757932

[B61] Zhang H. Hu H. Zhang X. B. Wang K. L. Song T. Q. Zeng F. P. (2012). Detecting Suaeda salsa L. chlorophyll fluorescence response to salinity stress by using hyperspectral reflectance. Acta Physiol. Plant. 34, 581–588. doi: 10.1007/s11738-011-0857-y

[B62] Zhang L. Ma H. J. Chen T. T. Pen J. Yu S. X. Zhao X. H. (2014). Morphological and physiological responses of cotton (Gossypium hirsutum L.) plants to salinity. PloS One 9, e112807. doi: 10.1371/journal.pone.0112807 25391141 PMC4229235

[B64] Zhang J. Y. Sun H. Gao D. H. Qiao L. Liu N. Li M. Z.. (2020). Detection of canopy chlorophyll content of corn based on continuous wavelet transform analysis. Remote Sens. 12, 2741–2741. doi: 10.3390/rs12172741

[B63] Zhang X. X. Yin X. L. Li H. L. Su D. Jia S. Y. Dong Z. (2017). Effect of NaCl stress on biomass and photosynthesis of different white elm lines. Acta Ecol. Sinica 37, 7258–7265. doi: 10.5846/stxb201608091632

[B66] Zhao R. M. An L. L. Song D. Li M. Z. Qiao L. Sun H. (2021). Detection of chlorophyll fluorescence parameters of potato leaves based on continuous wavelet transform and spectral analysis. Spectrochimica Acta Part A: Mol. Biomol. Spectrosc. 259, 119768–119768. doi: 10.1016/J.SAA.2021.119768 33971438

[B67] Zheng W. Lu X. Li Y. Li S. Zhang Y. Z. (2021). Hyperspectral identification of chlorophyll fluorescence parameters of suaeda salsa in coastal wetlands. Remote Sens. 13, 2066. doi: 10.3390/RS13112066

[B69] Zhu C. M. Ding J. L. Zhang Z. P. Wang J. J. Chen X. Y. Han L. J.. (2023). Soil salinity dynamics in arid oases during irrigated and non-irrigated seasons. Land Degrad/ Dev. 34, 3823–3835. doi: 10.1002/ldr.4632

[B68] Zhu Y. Tian Y. C. Ma J. F. Yao X. Liu X. J. Cao W. X. (2007). Relationship between chlorophyll fluorescence parameters and reflectance spectrum characteristics of wheat leaves. Acta Agronomica Sinica 8, 1286–1292. doi: 10.3321/j.issn:0496-3490.2007.08.011

