# Supplementary material for: Improving the estimation accuracy of rapeseed leaf photosynthetic characteristics under salinity stress using continuous wavelet transform and successive projections algorithm
Source: Front Plant Sci. 2023 Nov 14;14:1284172. doi: 10.3389/fpls.2023.1284172 (PMC10733793; doi:10.3389/fpls.2023.1284172)
Supplement: Supplementary file 1 [file DataSheet_1.zip › Supplementary_Material/Supplementary_ Figures.docx]

Supplementary Figures





**Figure S1** Reflectance curves for the raw spectral data, standard normal variate-preprocessed spectral data, and different-scale continuous wavelet transformation-preprocessed spectral data


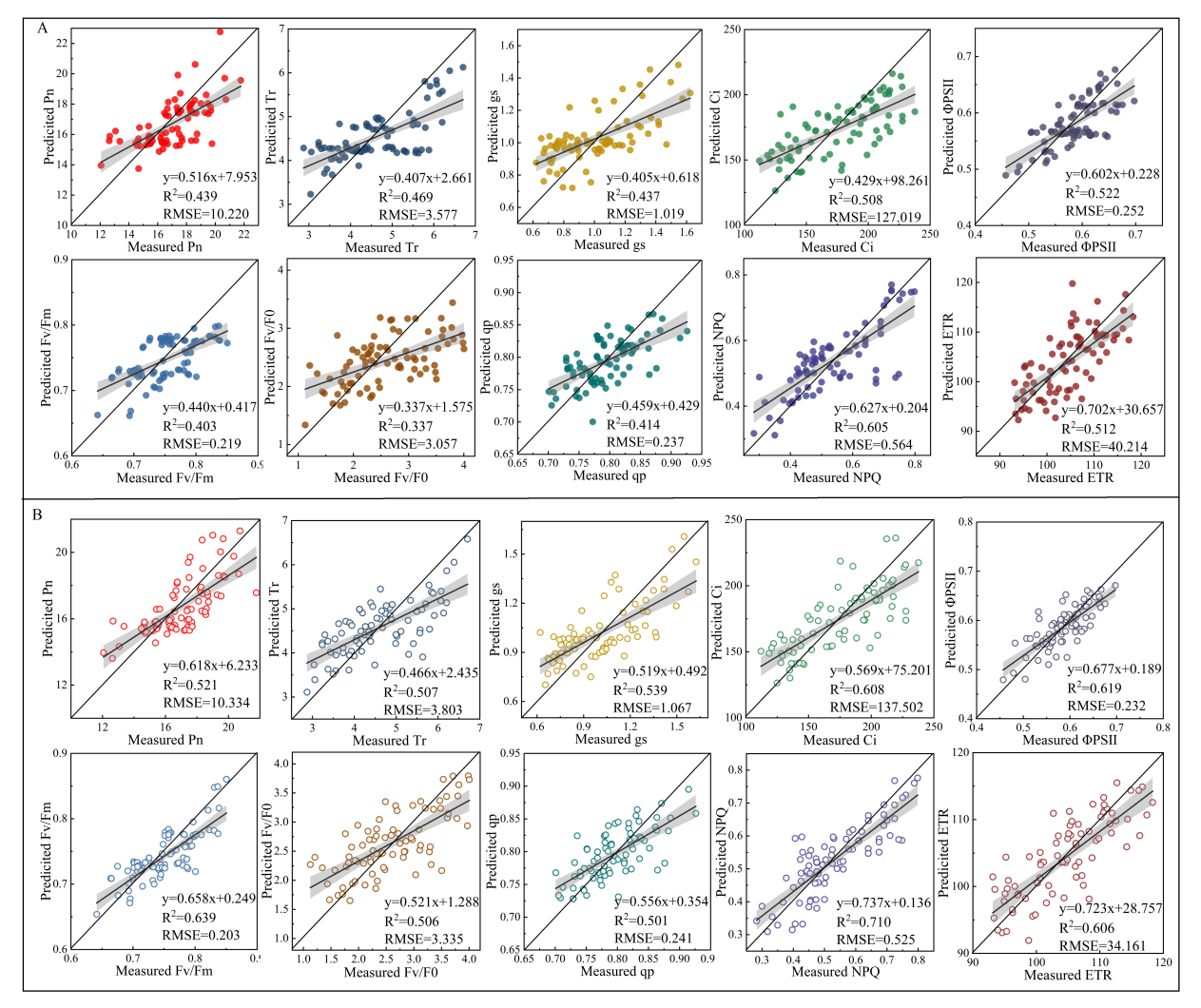


**Figure S2.** Model validation (n = 80). A is the validation of the accuracy of the model constructed based on the spectral features extracted from the red region in estimating photosynthetic gas exchange and chlorophyll fluorescence parameters of rapeseed under 10-day salinity stress, and B is the validation of the accuracy of the model constructed based on the spectral features extracted from full band in estimating photosynthetic gas exchange and chlorophyll fluorescence parameters of rapeseed under 10-day salinity stress.


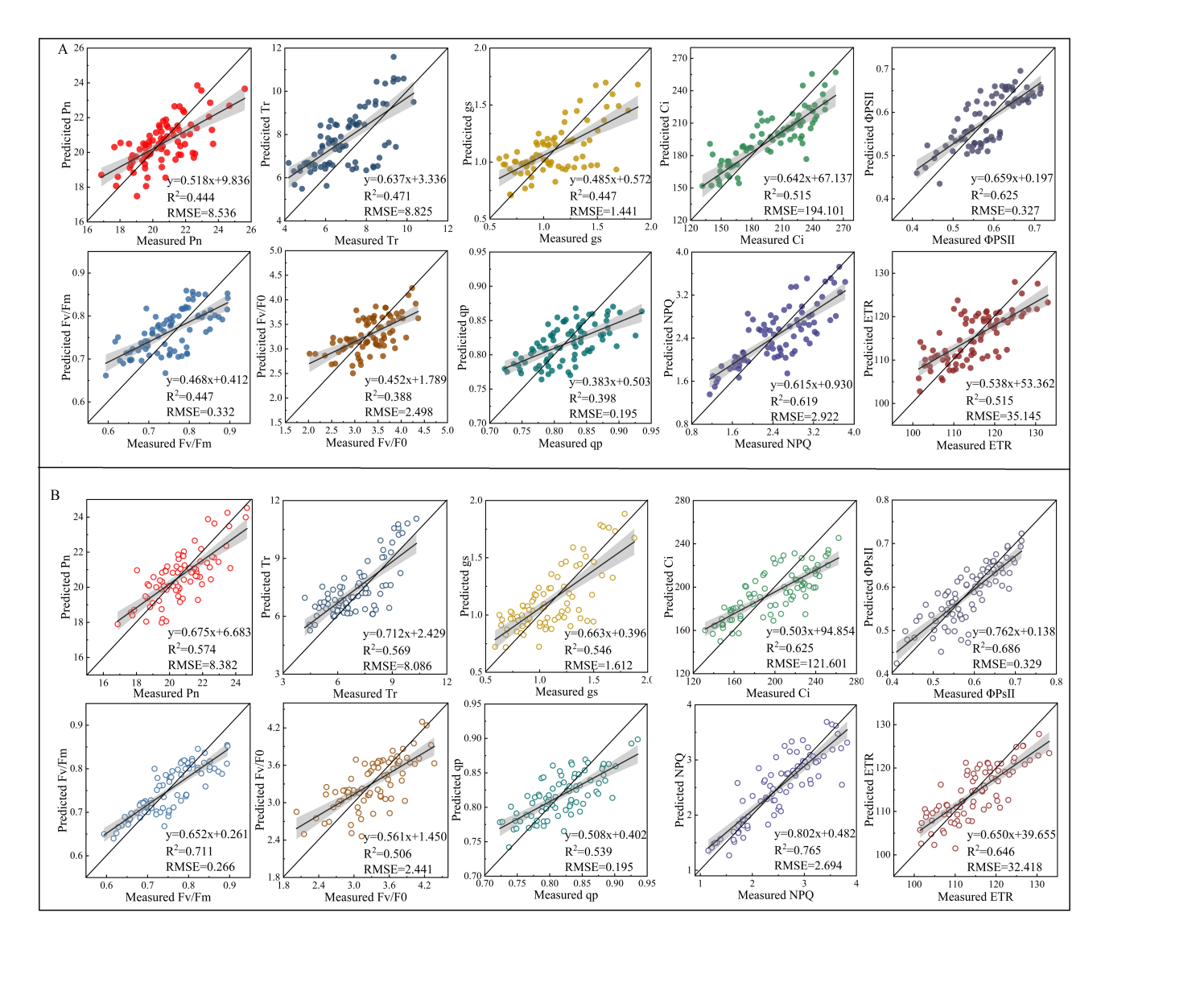


**Figure S3.** Model validation (n = 80). A is the validation of the accuracy of the model constructed based on the spectral features extracted from the red region in estimating photosynthetic gas exchange and chlorophyll fluorescence parameters of rapeseed under 20-day salinity stress, and B is the validation of the accuracy of the model constructed based on the spectral features extracted from full band in estimating photosynthetic gas exchange and chlorophyll fluorescence parameters of rapeseed under 20-day salinity stress.


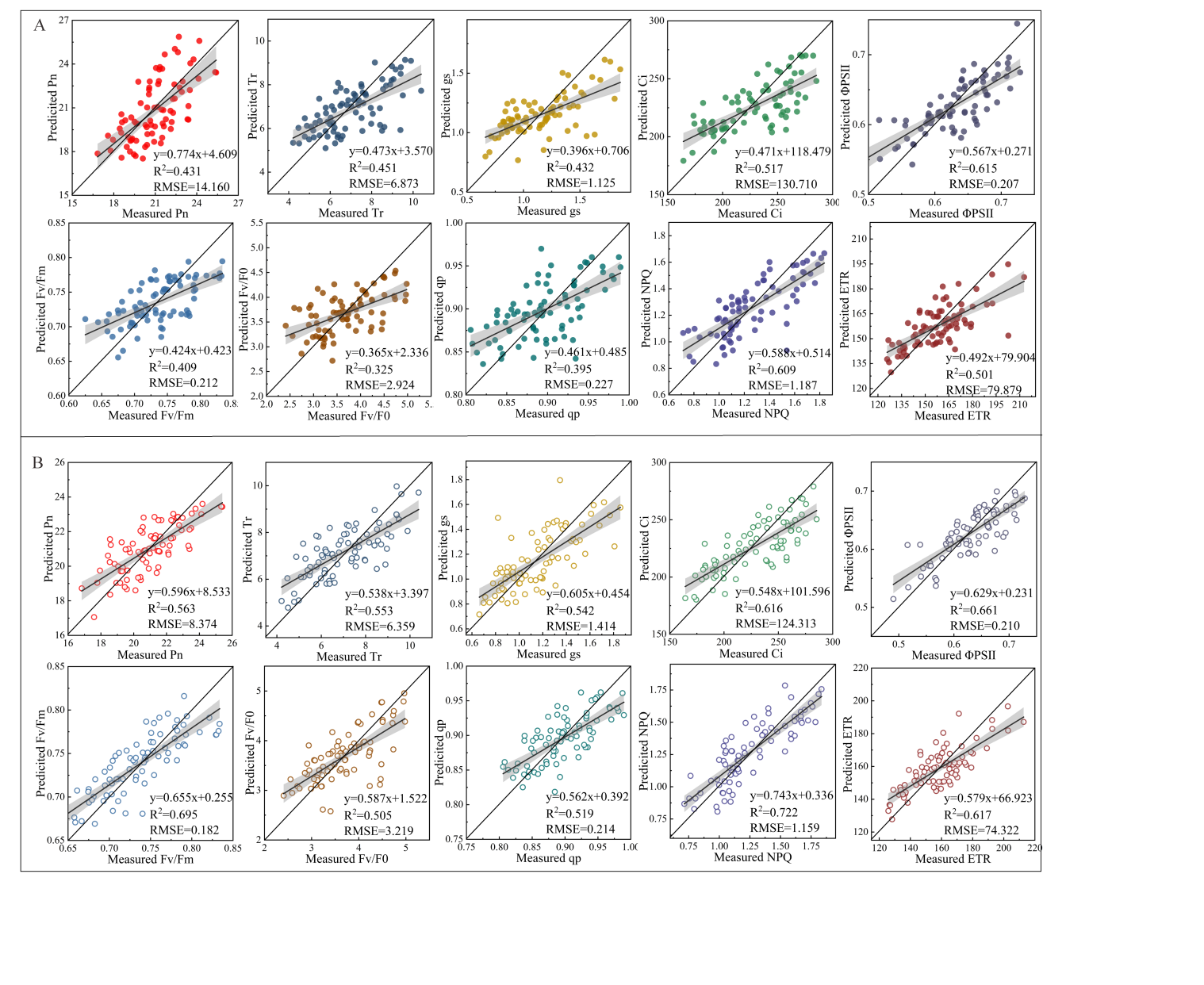


**Figure S4.** Model validation (n = 80). A is the validation of the accuracy of the model constructed based on the spectral features extracted from the red region in estimating photosynthetic gas exchange and chlorophyll fluorescence parameters of rapeseed under 40-day salinity stress, and B is the validation of the accuracy of the model constructed based on the spectral features extracted from full band in estimating photosynthetic gas exchange and chlorophyll fluorescence parameters of rapeseed under 40-day salinity stress.
