# Supplementary material for: Improving the estimation accuracy of rapeseed leaf photosynthetic characteristics under salinity stress using continuous wavelet transform and successive projections algorithm
Source: Front Plant Sci. 2023 Nov 14;14:1284172. doi: 10.3389/fpls.2023.1284172 (PMC10733793; doi:10.3389/fpls.2023.1284172)
Supplement: Supplementary file 1 [file DataSheet_1.zip › Supplementary_Material/Supplementary_Table.docx]

Supplementary Table

**Table S1.** Data set for the construction of the photosynthetic gas exchange and chlorophyll fluorescence parameter estimation model

| Item | 10 d | | | 20 d | | | 30 d | | | 40 d | | |
| --- | --- | --- | --- | --- | --- | --- | --- | --- | --- | --- | --- | --- |
|  | Total number | Modeling set | Validation set | Total number | Modeling set | Validation set | Total number | Modeling set | Validation set | Total number | Modeling set | Validation set |
| Pn | 238 | 159 | 79 | 240 | 160 | 80 | 239 | 159 | 80 | 240 | 160 | 80 |
| Tr | 240 | 160 | 80 | 240 | 160 | 80 | 240 | 160 | 80 | 240 | 160 | 80 |
| gs | 240 | 160 | 80 | 240 | 160 | 80 | 240 | 160 | 80 | 240 | 160 | 80 |
| Ci | 238 | 159 | 79 | 239 | 159 | 80 | 239 | 159 | 80 | 240 | 160 | 80 |
| ΦpsⅡ | 240 | 160 | 80 | 240 | 160 | 80 | 240 | 160 | 80 | 240 | 160 | 80 |
| fv/fm | 240 | 160 | 80 | 240 | 160 | 80 | 240 | 160 | 80 | 240 | 160 | 80 |
| fv/f0 | 240 | 160 | 80 | 240 | 160 | 80 | 240 | 160 | 80 | 240 | 160 | 80 |
| qP | 240 | 160 | 80 | 239 | 159 | 80 | 240 | 160 | 80 | 240 | 160 | 80 |
| NPQ | 240 | 160 | 80 | 240 | 160 | 80 | 240 | 160 | 80 | 240 | 160 | 80 |
| ETR | 240 | 160 | 80 | 240 | 160 | 80 | 239 | 159 | 80 | 239 | 159 | 80 |

Pn, net photosynthetic rate; Ci, intercellular carbon dioxide concentration; gs, stomatal conductance; Tr, transpiration rate; qP, quenching coefficient; NPQ, non-photochemical quenching coefficient; ΦPSII, actual photochemical efficiency of PSII; Fv/Fm, maximum photochemical efficiency of PSII; Fv/F0, potential activity of PSII; ETR, electron transport rate.
